# Virtual Observation Using Location-Dependent Statistical Information of Cyclists’ Movement for Estimation of Position and Uncertainty

**DOI:** 10.3390/s25165122

**Published:** 2025-08-18

**Authors:** Kento Suzuki, Takuma Ito

**Affiliations:** Graduate School of Engineering, The University of Tokyo, 7-3-1 Hongo, Tokyo 113-8656, Japan; suzuki-kento268@g.ecc.u-tokyo.ac.jp

**Keywords:** movement estimation, movement characteristics, Kalman filter, Kalman smoother, GNSS, mobility, intelligent transportation system

## Abstract

Crossing collisions between cyclists and automobiles around nonsignalized intersections on community roads, where visibility around the intersection is poor due to occlusions caused by house walls, is a social issue related to traffic safety in Japan. Because available observation information for collision prevention is limited on community roads, utilizing the accumulated data is useful to compensate for the lack of observation information. Given these motivations, we propose a movement estimation method of cyclists by combining information from roadside sensors with location-dependent statistical information. First, we develop a method for analyzing the location-dependent statistical information of cyclists on a certain road from accumulated GNSS data using the Kalman smoother. Then, we develop a method for stochastically predicting the movement of cyclists even outside the observation range of a roadside sensor by using the concept of “virtual observation” based on location-dependent statistical information. To evaluate the proposed method, we conduct an experiment to accumulate GNSS data from cyclists using smartphones. As a result of comparison with a conventional method, we confirm that our proposed method can reduce the uncertainty of the estimated position; further, the reduction in the uncertainty will contribute to traffic safety by future advanced driver assistance systems.

## 1. Introduction

**Figure 1:**
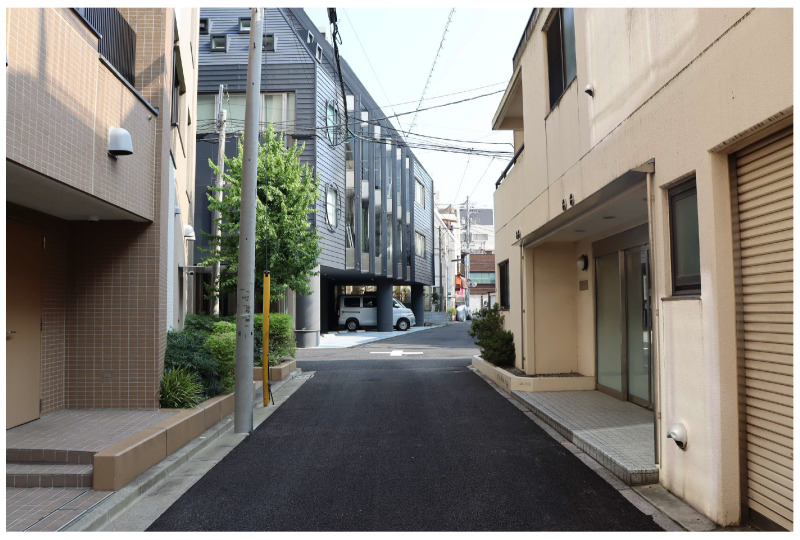
Appearance of a nonsignalized intersection on a community road in Japan.

Recently, the number of fatal traffic accidents in Japan has basically been declining [1]. However, the declining trend in the number of fatal traffic accidents on community roads is slow compared with that on non-community roads [2]. Figure 1 shows an example of a nonsignalized intersection on a community road. As shown in Figure 1, visibility around intersections on community roads is limited by narrow roads and building walls. Therefore, the risk of crossing collisions around such intersections is high. In fact, 45% of traffic accidents involving cyclists in Japan occur around intersections [3]. In addition, the velocity of cyclists is relatively high among vulnerable road users (VRUs), and cyclist-related traffic accidents are associated with a higher risk of severe damage. In summary, countermeasures are required to prevent cyclist-related collisions on community roads. Furthermore, this is not a problem specific to Japan, but a problem found in other regions as well. For example, cyclist fatalities are decreasing only slightly in the EU [4], and a high percentage of cyclist fatalities occur at intersections compared to other traffic modes [4]. Additionally, the authors of [5] pointed out that traffic accidents related to cyclists and electric bike riders are a current social problem in China. Thus, the countermeasures are also required in other regions.

**Figure 2:**
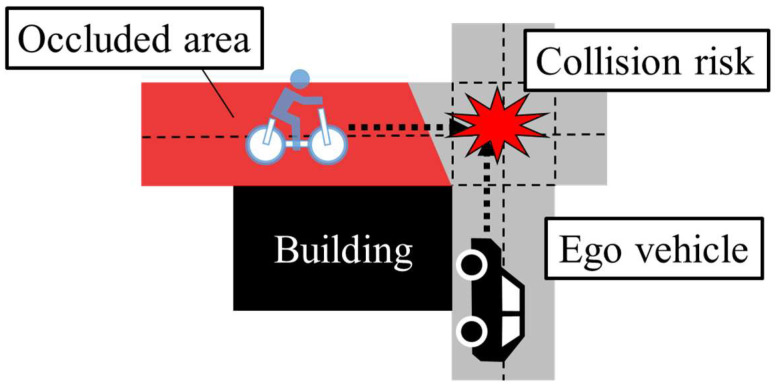
Conceptual schematic of a cyclist moving behind occlusions.

To prevent cyclist-related crossing collisions, it is important to grasp the position of the cyclists moving on intersecting roads, as shown in Figure 2. Because current advanced driver assistance systems (ADASs) for commercial vehicles mainly use onboard sensors, they may not work adequately in road environments with occlusions, such as community roads. In such environments, the placement of roadside sensors is an effective approach for reliably preventing traffic accidents. However, the placement of roadside sensors at every intersection is not practical from a cost perspective. In another approach, although using real-time position information provided by portable devices, such as smartphones, is also effective, not all cyclists provide their position information. Thus, the challenge in preventing collisions on community roads is that the available real-time information is limited.

**Figure 3:**
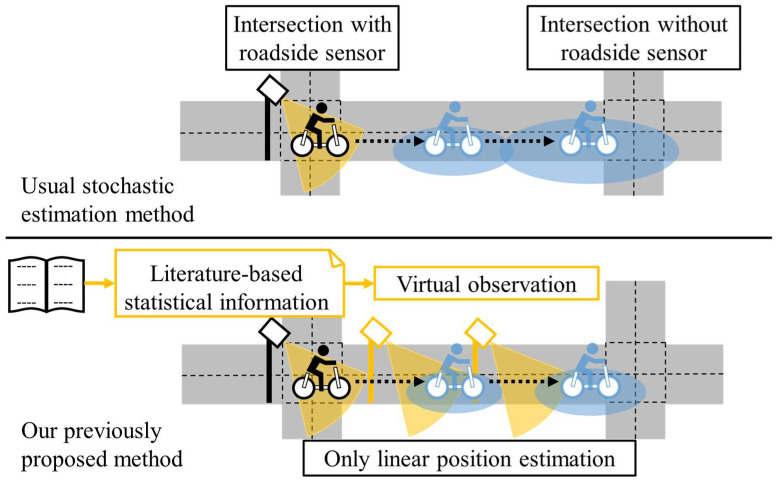
Conceptual schematic of assumed situation and our previously proposed method.

Figure 3 shows a conceptual schematic of the traffic environment assumed in this study. An important point is that roadside sensors are placed sparsely; some intersections have roadside sensors, whereas others do not. More precisely, we assume a situation where a cyclist moves from the first intersection, which has a roadside sensor, to the second intersection, which does not have roadside sensors. Although the distance between intersections varies depending on the town, we assume 100 m as a baseline of the relatively long distance between intersections in Japan. To prevent crossing collisions around the second intersection, it is necessary to estimate the position and uncertainty of the cyclist via a stochastic position estimation method using previously observed information around the first intersection. From the viewpoint of the prevention of crossing collisions, smaller uncertainty of the estimated position is desirable because connected ADASs can behave more adequately using the estimated position with less uncertainty [6]. In the usual stochastic position estimation method, the uncertainty of the position estimation increases if the estimation system lacks observation information. Thus, the challenge in the assumed situation of this study is that the uncertainty is large because of the lack of real-time observations. However, although the movement of cyclists between intersections cannot be observed in the assumed situation, we can roughly estimate their movement based on statistical information. With these motivations, in our previous study [7], we proposed a method for compensating insufficient information of real-time observation by the concept of “virtual observation” based on literature-based statistical information, such as average velocity, and confirmed that this approach could contribute to adequate position estimation by reducing the uncertainty of the position estimation.

**Figure 4:**
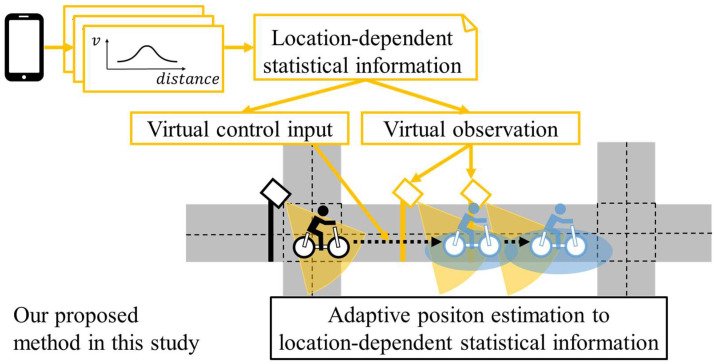
Conceptual schematic of the proposed method in this study.

However, because literature-based statistical information describes only the average movement, we assumed only static movement without acceleration/deceleration in the previous study. Consequently, the generality of the proposed method was limited. Thus, considering dynamic motion is necessary to extend the method further. Because acceleration/deceleration of cyclists occurs due to the elements of the road environment, such as intersections and crosswalks, such dynamic movements depend on the location. Thus, if we accumulate and analyze the movement data of cyclists related to a certain road, we can estimate location-dependent movement on the road, even under the assumption that cyclists accelerate or decelerate. From the viewpoint of stochastic motion estimation methods, such as the Kalman filter, such dynamic motion information is usually described as a control input. Thus, we call the assumed location-dependent dynamic movement related to acceleration and yawing rate as “virtual control input.” Figure 4 summarizes a conceptual schematic of our newly proposed method in this study.

Given the above motivations, in this study, we propose a method for analyzing such location-dependent statistical information (LDSI) by considering the differences among locations and individuals, as well as a method for predicting the position and uncertainty of cyclists based on LDSI. First, we propose a Kalman-smoother-based method for analyzing movement characteristics using GNSS data which was obtained and accumulated from cyclists’ smartphones. Then, we cluster the data by considering the movement characteristics that depend on the location and individual characteristics. These processes allow us to prepare LDSI from accumulated data. Subsequently, we propose a method for estimating the position and uncertainty of cyclists based on LDSI. The main contributions of this study are as follows:

- We propose a method for analyzing LDSI from accumulated low-level GNSS data.
- We propose a method for predicting the position and uncertainty of cyclists via virtual observation and virtual control input based on the LDSI.

In this study, we verified the proposed methods by the simulation using the data obtained in the experiments under the control conditions. In other words, the position estimation method was not verified by the actual experiment in the real-world community roads, but only by the simulation.

The remainder of this paper is organized as follows. Section 2 presents related works on traffic safety technologies. The proposed method is described in detail in Section 3. Section 4 describes the experiments for data accumulation and the analysis results of LDSI preparation. Section 5 describes the simulation experiment of movement prediction using LDSI. Finally, Section 6 concludes the study and describes future work.

## 2. Related Works

This study aims to develop technologies related to cooperative safety systems to prevent traffic accidents around nonsignalized intersections with occlusions. Thus, Section 2.1 organizes existing studies related to cooperative perception methods. Additionally, cooperative safety systems require the position estimation of other traffic participants, considering the movement characteristics that depend on individual and location characteristics. Therefore, Section 2.2 organizes the existing studies related to the position estimation method considering movement characteristics based on accumulated data. Finally, Section 2.3 organizes existing studies on position estimation using technologies related to virtual observations and virtual control inputs.

### 2.1. Cooperative Perception Methods for Traffic Safety of Vulnerable Road Users

Estimating the positions of other traffic participants is necessary to prevent traffic accidents in road environments with occlusions. Existing studies related to the position estimation of VRUs are roughly classified into two approaches: methods using roadside units (RSUs) that are equipped with various sensors, and methods using portable devices such as smartphones.

As for the methods using RSUs, various types of sensors have been utilized. Bo-Bo et al. [8] proposed a traffic participant tracking method that uses multiple cameras installed on RSUs. Zhang et al. [9] proposed an improved method for vehicle velocity observation using roadside LiDAR. Zhang et al. [10] installed fisheye cameras in a roundabout and verified their performance in terms of vehicle perception.

As for the methods using portable devices, smartphones have been frequently utilized. Sugimoto et al. [11] developed a collision warning system which utilizes V2P (Vehicle to Pedestrian) communication via a cellular phone and car navigation system. Liu et al. [12] developed Vehicle to Infrastructure (V2I)/Pedestrian to Infrastructure (P2I) communication devices and a collision warning application that uses observation information from a roadside radar. Hussein et al. [13] proposed a collision warning application and evaluated its usability.

Both approaches are useful in preventing traffic accidents. However, because placing many RSUs so densely that all traffic participants can always be observed is costly, this approach is difficult, especially on community roads. Additionally, not all traffic participants are willing to provide their position information from their portable devices. Thus, we need to design a new method based on the assumption that information only from sparsely placed RSU and information from portable devices of a limited number of cyclists can be used. Therefore, we design a new method that prepares LDSI from data which is obtained and accumulated from a limited number of cyclists, and estimates the position of cyclists by combining information from sparsely placed RSUs with LDSI.

### 2.2. Position Estimation Methods Considering Movement Characteristics Based on Accumulated Data

The movement characteristics of cyclists depend on various factors such as traffic volume, road width, and the existence of crosswalks. Thus, the use of LDSI prepared from accumulated data on each road is effective for predicting the positions of cyclists. Related to data-driven prediction methods, existing studies have proposed two approaches: methods using machine learning (ML), which do not clearly define the motion model, and methods that use motion models with adjusting the model parameters.

As an example of the methods using ML, Li et al. [14] utilized Gaussian process regression models to predict the trajectories of traffic participants which were observed by roadside sensors. Zhou et al. [15] proposed a method using long short-term memory (LSTM) to estimate pedestrian trajectories and collision risk. Deo et al. [16] proposed an LSTM encoder–decoder model that uses convolutional social pooling to predict vehicle trajectories, considering the interaction between vehicles. Liu et al. [17] proposed a Transformer-based method that predicts vehicle trajectories multimodally. Liu’s method used previous trajectories of observed vehicles and map information for prediction.

On the other hand, methods using motion models based on accumulated data contrast with the above ones. Such methods analyzed the accumulated data and then adjusted parameters related to the estimation model, such as the process noise matrix and measurement noise matrix in the extended Kalman filter (EKF). For instance, Boulkroune et al. [18] proposed a tuning method for the process noise matrix and measurement noise matrix of the EKF. They tuned the matrices by minimizing the cost function in the sample data. Jouaber et al. [19] tuned the process noise matrix of the EKF in real-time estimation using a recurrent neural network (RNN). In Jouaber’s method, they trained an RNN-based model, which utilizes the innovation as an input and calculates the process noise matrix, using accumulated trajectories. Additionally, some studies clustered accumulated data and utilized the results for the position estimation and intention prediction of traffic participants. For instance, Jeong et al. [20] collected driving data and clustered the data by velocity. Subsequently, they defined different EKF models for each cluster and utilized them for real-time trajectory prediction. Yi et al. [21] collected vehicles’ trajectory data at a certain intersection and conducted a polynomial regression mixture clustering algorithm. Subsequently, they trained the classification and regression tree algorithm and predicted the drivers’ behavior.

Because the former approach using ML does not explicitly model the motion of the targets, it cannot use the observation information from sensors whose information is not learned by the ML model. Thus, we select the latter approach, which defines the motion model and adjusts its parameters.

### 2.3. Virtual Observations and Virtual Control Inputs for Kalman Filter

As methods similar to our proposed method in this study, we organize existing studies related to the Kalman filter and virtual observation. Tahk et al. [22], and Alouani et al. [23] proposed methods for estimating the position of airplanes using virtual observations. In their method, movement constraints were utilized as virtual observations. Because the movement of ground vehicles is constrained by the road, virtual observations derived from such constraints have been utilized in some research. Zhou et al. [24] utilized the trajectory shape constraint as a virtual observation. In addition, Zhang et al. [25] proposed a position estimation method that utilizes heading direction constraints derived from road geometry.

Furthermore, some studies have derived virtual observations using ML-based methods. Vargas-Meléndez et al. [26] estimated the roll angle of vehicles by using virtual observation derived by a neural network (NN) module. Kim et al. [27] proposed a method that uses virtual observations derived by an LSTM-based network to estimate the sideslip angle of a vehicle. In the methods above, virtual observations are calculated from actual sensor observations using ML methods.

In addition to virtual observations, some researchers have investigated the utilization of virtual control inputs. Jiang et al. [28] utilized an NN-based module to derive virtual control input and process noise covariance and estimated the position of the aircraft using the EKF. Kim et al. [27] utilized the angular velocity derived by an LSTM-based network as a virtual control input, in addition to virtual observation.

The above methods improve the estimation accuracy by additionally inputting supplemental information, such as virtual observation and virtual control input, only when the methods obtain some observation information. Thus, these methods cannot compensate for the continuous lack of real-time information. Based on this background, in our previous study, we proposed a method that assumes virtual observation information from literature-based statistical information to improve movement estimation [7]. As a result of numerical simulations and experiments in the real world, we confirmed that our previous method adequately estimated the position using virtual observations. However, in our previous study, we used only literature-based statistical information and did not discuss how to prepare data-driven statistical information. In addition, we assumed that the virtual observation information was always constant and did not discuss the locationally adaptive method using LDSI. Thus, in this study, we propose new methods that prepares LDSI and predicts the position of cyclists using LDSI. Furthermore, we discuss a new approach that uses a virtual control input, which was not used in our previous study.

## 3. Method

### 3.1. Investigated Situation

**Figure 5:**
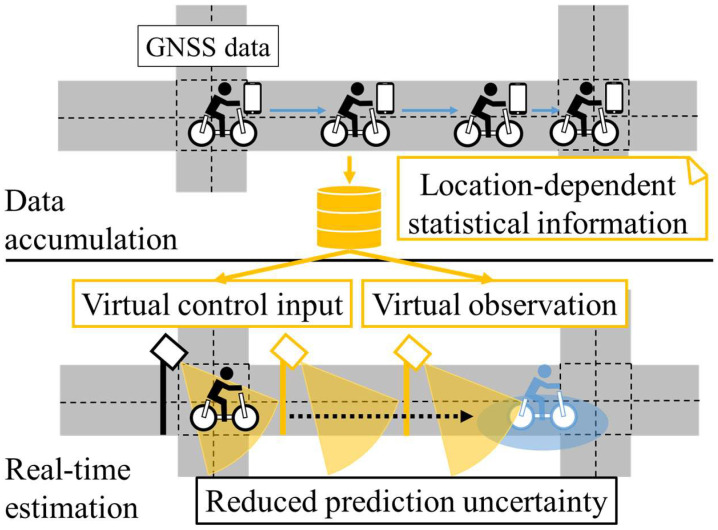
Conceptual schematic of LDSI preparation and application. The details of analysis in the data accumulation and analysis in the real-time estimation are explained in Section 3.3 and Section 3.4, respectively. The details of the experiment related to the data accumulation and the numerical simulation related to the real-time estimation are explained in Section 4 and Section 5, respectively.

In this study, we analyze the movement characteristics of cyclists from the accumulated data of low-level GNSS obtained by smartphones, and then prepare the LDSI. After that, we utilize the LDSI for position estimation. Figure 5 shows a conceptual schematic of the proposed method. The mean and standard deviation of the movement state variables at each location are utilized as the LDSI. LDSI enables us to improve the accuracy of movement prediction of a cyclist observed by an RSU, even if real-time GNSS data of the cyclist cannot be obtained. Here, the important point is that the data from cyclists who agree to provide their GNSS data improves the estimation accuracy for cyclists who do not provide GNSS data.

### 3.2. Conceptual Design

**Figure 6:**
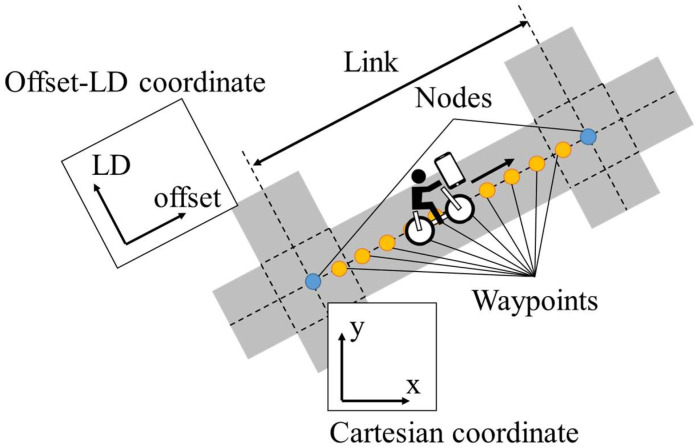
Conceptual schematic of the node-link map assumed in this study.

To use location-dependent information, we must associate the accumulated information with digital map systems. Although many existing studies have proposed various digital map systems, we assume a relatively simple digital map system consisting of nodes and links that correspond to intersections and roads between intersections in the real world. Figure 6 shows a conceptual schematic of the digital map system. In such a digital map system, links contain waypoints that are placed at a certain interval along the road, and the position of each waypoint is expressed by the distance along the road. In this study, because we assume the interval of waypoints to be 1 m, the spatial resolution of the LDSI associated with each waypoint is also 1 m. Additionally, we call the distance as “offset” by referring to the digital map system that we developed in our previous study [29]. Similarly, we call the lateral deviation perpendicular to the road direction as “LD” (lateral deviation).

**Figure 7:**
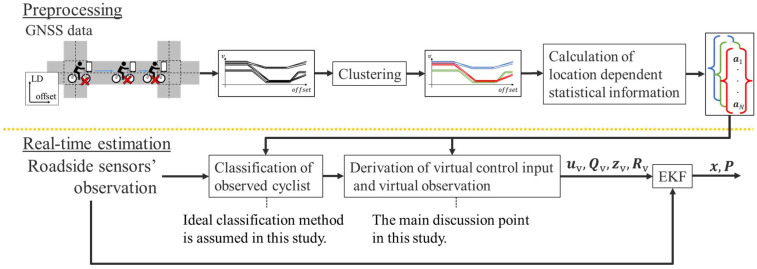
Overview of the proposed method. The red cross signs in the top left figure indicate the position of cyclists. The top middle graphs indicate the velocity along offset axis. The colors of the graph indicate the corresponding clusters. The top right vectors indicate LDSI of clusters. The methods related to preprocessing and real-time estimation are explained in Section 3.3 and Section 3.4, respectively.

Figure 7 shows an overview of the proposed method. As shown in Figure 7, the proposed method is divided into two parts: preprocessing and real-time estimation. The preprocessing part analyzes LDSI from accumulated GNSS data, and the real-time estimation part by EKF combines LDSI with roadside sensor observations for movement estimation of cyclists.

In the preprocessing part, we first accumulate GNSS data from portable devices such as smartphones on a certain road. By analyzing the accumulated GNSS data with a Kalman smoother [30], we estimate the movement state of cyclists with their uncertainties. Although the Kalman smoother estimates the time-series movement state, the movement characteristics of cyclists depend on each location. Thus, we spatially resample the estimated state at each waypoint. Subsequently, we cluster the series of resampled states by considering the estimated states and their uncertainties. Finally, we derive the LDSI for each cluster from the resampled states.

In real-time estimation part, the process consists of three components: classification of observed cyclist, derivation of virtual control input and virtual observation, and EKF for position estimation. First, a classification method of the cyclists observed by the roadside sensor for an appropriate cluster, which are predefined in the preprocessing step, is required. However, the main purpose of this study is to propose a method to utilize LDSI. Thus, in this study, we assume an ideally accurate classification method and focus on the following part. As for the assumption of the classification method, we will develop the method which classifies cyclists by comparing the cyclists’ movement states, such as velocity and spatial difference of velocity, estimated by roadside sensor with LDSI in future studies.

Next, the current estimated position is used to obtain the reference waypoint for the derivation of the virtual observation and virtual control input from the LDSI. Here, it is important to note that the current position is estimated with its uncertainty. Therefore, the choice of reference waypoints also requires consideration of uncertainty. Thus, we design a method to derive virtual observations and virtual control inputs, considering such uncertainty. The detailed method is described in Section 3.4.3.

Finally, by integrating the information of the virtual observation and virtual control input with the real roadside sensor’s observation, we estimate the cyclist’s movement state. Here, the LDSI related to the directional angle and velocity are utilized as the virtual observation, and the LDSI related to the yawing rate and acceleration are utilized as virtual control inputs. In addition, the variance of these states in clustered data is utilized as the uncertainty of the prediction and observation of EKF.

### 3.3. Preparation of LDSI

#### 3.3.1. Movement State Estimation Using Kalman Smoother

As mentioned in the previous section, we utilize the accumulated GNSS data of cyclists in the preprocessing part. Because the position obtained by GNSS contains an error, a method for handling this error is required. The movement state of cyclists are not analyzed in real time, but after accumulation. Thus, we utilize the Kalman smoother, which can be used only in post analysis and can estimate the movement state with smaller uncertainty than the Kalman filter. More precisely, in this study, we utilize a fixed-interval smoothing algorithm [30] for the Kalman smoother. The forward-direction filter is first applied in the fixed-interval smoothing algorithm. Subsequently, a backward-direction filter is applied. The forward-direction filter consists of a normal EKF.

Although the Kalman smoother and EKF are widely used in existing studies, we explain the equations of the Kalman smoother and EKF in the following paragraphs to clarify the state variables and motion models used in this study and their relations. In the usual EKF for estimating a cyclist’s movement state, the planar positions, directional angle, and velocity are often expressed as state variables. On the other hand, yawing rate and acceleration are often utilized as control inputs. However, the analysis for LDSI needs to estimate the yawing rate and acceleration in addition to other variables. Thus, in the analysis using the Kalman smoother, the yawing rate and acceleration are also expressed as state variables instead of control inputs, and the state variable $x_{S}$ and estimation covariance matrix $P_{S}$ are as follows:

(1) $$x_{S}={\left(\begin{array}{cccccc} x & y & \theta & v & \omega & a \end{array}\right)}^{T}$$

(2) $$P_{S}=\left(\begin{array}{cccccc} \sigma_{x}^{2} & \sigma_{xy} & \sigma_{x\theta } & \sigma_{xv} & \sigma_{x\omega } & \sigma_{xa}\\ \sigma_{xy} & \sigma_{y}^{2} & \sigma_{y\theta } & \sigma_{yv} & \sigma_{y\omega } & \sigma_{ya}\\ \sigma_{x\theta } & \sigma_{y\theta } & \sigma_{\theta }^{2} & \sigma_{\theta v} & \sigma_{\theta \omega } & \sigma_{\theta a}\\ \sigma_{xv} & \sigma_{yv} & \sigma_{\theta v} & \sigma_{v}^{2} & \sigma_{v\omega } & \sigma_{va}\\ \sigma_{x\omega } & \sigma_{y\omega } & \sigma_{\theta \omega } & \sigma_{v\omega } & \sigma_{\omega }^{2} & \sigma_{\omega a}\\ \sigma_{xa} & \sigma_{ya} & \sigma_{\theta a} & \sigma_{va} & \sigma_{\omega a} & \sigma_{a}^{2} \end{array}\right)$$

where x and y denote the planar positions in the Cartesian coordinate system based on the East and North directions, θ denotes the directional angle, v denotes the velocity, ω denotes the yawing rate, and a denotes the acceleration.

The EKF equations in the forward-direction filter are expressed as follows:

(3) $$\begin{array}{c} x_{S}\left(t_{k}\right)=f_{S}(x_{S}\left(t_{k-1}\right),n_{S}\left(t_{k-1}\right)) \end{array}$$

(4) $$z_{S}\left(t_{k}\right)=h_{S}(x_{S}\left(t_{k}\right))$$

where $t_{k}$ denotes the discretized time, $n_{S}$ denotes the noise, $f_{S}$ denotes the state transition function, $z_{S}$ denotes the observation, and $h_{S}$ denotes the observation function. The EKF consists of two parts: prediction and update. As for the prediction of EKF, the formulas are expressed as follows:

(5) $${\hat{x}}_{S}\left(t_{k}|t_{k-1}\right)=f_{S}\left({\hat{x}}_{S}\left(t_{k-1}|t_{k-1}\right),0\right)$$

(6) $$P_{S}\left(t_{k}|t_{k-1}\right)=F_{S}\left(t_{k-1}\right)P_{S}\left(t_{k-1}|t_{k-1}\right)F_{S}^{T}\left(t_{k-1}\right)+\Gamma_{S}\left(t_{k-1}\right)Q_{S}\Gamma_{S}^{T}\left(t_{k-1}\right)$$

where ${\hat{x}}_{S}$ denotes the filtered state, $F_{S}$ denotes the matrix of the partial derivatives of $f_{S}$ by $x_{S}$ , $\Gamma_{S}$ denotes the matrix of the partial derivatives of $f_{S}$ by $n_{S}$ , and $Q_{S}$ denotes the process noise matrix. As for the update of EKF, the formulas are expressed as follows:

(7) $$S_{S}\left(t_{k}|t_{k-1}\right)=H_{S}P_{S}\left(t_{k}|t_{k-1}\right)H_{S}^{T}+R_{S}$$

(8) $$K_{S}\left(t_{k}\right)=P_{S}\left(t_{k}|t_{k-1}\right)H_{S}^{T}S_{S}^{-1}\left(t_{k}|t_{k-1}\right)$$

(9) $${\hat{x}}_{S}\left(t_{k}|t_{k}\right)={\hat{x}}_{S}\left(t_{k}|t_{k-1}\right)+K_{S}\left(t_{k}\right)\left[z_{S}\left(t_{k}\right)-h_{S}\left({\hat{x}}_{S}\left(t_{k}|t_{k-1}\right)\right)\right]$$

(10) $$\begin{array}{c} P_{S}\left(t_{k}|t_{k}\right)=\left[I-K_{S}\left(t_{k}\right)H_{S}\right]P_{S}\left(t_{k}|t_{k-1}\right)\; \end{array}{\left[I-K_{S}\left(t_{k}\right)H_{S}\right]}^{T}+K_{S}\left(t_{k}\right)R_{S}K_{S}^{T}\left(t_{k}\right)$$

where $S_{S}$ denotes the system uncertainty matrix, $R_{S}$ denotes the measurement noise matrix, $K_{S}$ denotes the Kalman gain, and $H_{S}$ denotes the matrix of the partial derivatives of $h_{S}$ by $x_{S}$ . Here, we assume that the noise $n_{S}$ is applied to the yawing rate and acceleration. Therefore, the state transition function $f_{S}$ is expressed as follows:

(11) $$f_{S}\left(x_{S}\left(t_{k-1}\right),n_{S}\left(t_{k-1}\right)\right)=\left(\begin{array}{c} x\left(t_{k-1}\right)+v\left(t_{k-1}\right)∆t\cos\theta \left(t_{k-1}\right)+\frac{1}{2}a\left(t_{k-1}\right)∆t^{2}\cos\theta \left(t_{k-1}\right)+\frac{1}{2}a_{noise}∆t^{2}\cos\theta \left(t_{k-1}\right)\\ y\left(t_{k-1}\right)+v\left(t_{k-1}\right)∆t\sin\theta \left(t_{k-1}\right)+\frac{1}{2}a\left(t_{k-1}\right)∆t^{2}\sin\theta \left(t_{k-1}\right)+\frac{1}{2}a_{noise}∆t^{2}\sin\theta \left(t_{k-1}\right)\\ \theta \left(t_{k-1}\right)+\omega \left(t_{k-1}\right)∆t+\omega_{noise}∆t\\ v\left(t_{k-1}\right)+a\left(t_{k-1}\right)∆t+a_{noise}∆t\\ \omega \left(t_{k-1}\right)+\omega_{noise}\\ a\left(t_{k-1}\right)+a_{noise} \end{array}\right)$$

where ∆t denotes the time step between the predictions. Moreover, the noise $n_{S}$ and the process noise matrix $Q_{S}$ are as follows:

(12) $$n_{S}={\left(\begin{array}{cc} \omega_{noise} & a_{noise} \end{array}\right)}^{T}$$

(13) $$Q_{S}=\left(\begin{array}{cc} \sigma_{\omega \_noise}^{2} & 0\\ 0 & \sigma_{a\_noise}^{2} \end{array}\right)$$

where $\omega_{noise}$ denotes the noise of the yawing rate, $a_{noise}$ denotes the noise of acceleration, $\sigma_{\omega \_noise}$ denotes the standard deviation of $\omega_{noise}$ , and $\sigma_{a\_noise}$ denotes the standard deviation of $a_{noise}$ . From the above discussions, $F_{S}$ and $\Gamma_{S}$ are derived as follows:

(14) $$\begin{array}{cc} & F_{S}\left(t_{k-1}\right)=\frac{\partial f_{S}\left(x_{S},n_{S}\right)}{\partial x_{S}}{|}_{x_{S}=x_{S}\left(t_{k-1}\right),n_{S}=0}\\ & =\left(\begin{array}{cccccc} 1 & 0 & -v\left(t_{k-1}\right)∆t\sin\theta \left(t_{k-1}\right)-\frac{1}{2}a\left(t_{k-1}\right)∆t^{2}\sin\theta \left(t_{k-1}\right) & ∆t\cos\theta \left(t_{k-1}\right) & 0 & \frac{1}{2}∆t^{2}\cos\theta \left(t_{k-1}\right)\\ 0 & 1 & v\left(t_{k-1}\right)∆t\cos\theta \left(t_{k-1}\right)+\frac{1}{2}a\left(t_{k-1}\right)∆t^{2}\cos\theta \left(t_{k-1}\right) & ∆t\sin\theta \left(t_{k-1}\right) & 0 & \frac{1}{2}∆t^{2}\sin\theta \left(t_{k-1}\right)\\ 0 & 0 & 1 & 0 & ∆t & 0\\ 0 & 0 & 0 & 1 & 0 & ∆t\\ 0 & 0 & 0 & 0 & 1 & 0\\ 0 & 0 & 0 & 0 & 0 & 1 \end{array}\right) \end{array}$$

(15) $$\Gamma_{S}\left(t_{k-1}\right)=\frac{\partial f_{S}\left(x_{S},n_{S}\right)}{\partial n_{S}}{|}_{x_{S}=x_{S}\left(t_{k-1}\right)}=\left(\begin{array}{cc} 0 & \frac{1}{2}∆t^{2}\cos\theta \left(t_{k-1}\right)\\ 0 & \frac{1}{2}∆t^{2}\sin\theta \left(t_{k-1}\right)\\ ∆t & 0\\ 0 & ∆t\\ 1 & 0\\ 0 & 1 \end{array}\right)$$

As for $Q_{S}$ , we derive the parameters referring to the assumed maximum yawing rate and maximum acceleration. We assume that the maximum yawing rate is achieved when cyclists change their direction at a small intersection with an average velocity. In addition, we assume that maximum acceleration is achieved when the cyclists start moving. We derive these values from the road structure and cyclists’ general movements in Japan. From the standard value of corner cut length at low traffic intersections 3.0 m [31], and cyclists’ average velocity 15 km/h [32], the maximum yawing rate is calculated as follows:

(16) $$\omega_{max}=\frac{\pi }{2}÷\left(\frac{3.0\times \frac{\pi }{2}}{\frac{15\;\times \;1000}{3600}}\right)=1.38\;rad/s$$

From the maximum yawing rate above and the maximum acceleration of 1.95 m/s [33], $Q_{S}$ is determined as follows:

(17) $$Q_{S}=\left(\begin{array}{cc} {\left(\frac{\omega_{max}}{2}\right)}^{2} & 0\\ 0 & {\left(\frac{a_{max}}{2}\right)}^{2} \end{array}\right)=\left(\begin{array}{cc} 0.7^{2} & 0\\ 0 & 1.0^{2} \end{array}\right)$$

The remaining undetermined variables in the forward-direction filtering are $z_{S}$ , $H_{S}$ , and $R_{S}$ . Because these variables depend on the sensor characteristics, we consider them in Section 3.3.2.

After the forward-direction filtering, the backward filtering is conducted. Similarly to the forward-direction filtering, the backward filtering consists of two parts: prediction and update. The prediction part is expressed as follows:

(18) $$P_{S}^{\prime }\left(t_{k+1}|t_{k}\right)=F_{S}\left(t_{k}\right)P_{S}\left(t_{k}|t_{k}\right)F_{S}^{T}\left(t_{k}\right)+\Gamma_{S}\left(t_{k}\right)Q_{S}\Gamma_{S}^{T}\left(t_{k}\right)$$

where $P_{S}^{\prime }$ denotes the covariance matrix of smoothed state ${\hat{x}}_{S}^{\prime }$ . As for the update part, the formula is expressed as follows:

(19) $$K_{S}^{\prime }\left(t_{k}\right)=P_{S}\left(t_{k}|t_{k}\right)F_{S}^{T}\left(t_{k}\right){P_{S}^{\prime }}^{-1}\left(t_{k+1}|t_{k}\right)$$

(20) $${\hat{x}}_{S}^{\prime }\left(t_{k}|t_{k}\right)={\hat{x}}_{S}\left(t_{k}|t_{k}\right)+K_{S}^{\prime }\left(t_{k}\right)\left[{\hat{x}}_{S}^{\prime }\left(t_{k+1}|t_{k+1}\right)-f_{S}\left({\hat{x}}_{S}\left(t_{k}|t_{k}\right),0\right)\right]$$

(21) $$P_{S}^{\prime }\left(t_{k}|t_{k}\right)=P_{S}\left(t_{k}|t_{k}\right)+K_{S}^{\prime }\left(t_{k}\right)\left[P_{S}^{\prime }\left(t_{k+1}|t_{k+1}\right)-P_{S}^{\prime }\left(t_{k+1}|t_{k}\right)\right]{K_{S}^{\prime }}^{T}\left(t_{k}\right)$$

where $K_{S}^{\prime }$ denotes the smoother gain.

#### 3.3.2. Implementation of Observation

In this study, GNSS data are mainly assumed to be a candidate for accumulated data. However, observations by other sensors, such as roadside LiDAR, can be utilized when available. Because not all types of sensors can directly observe direction and velocity, we alternatively calculate the direction and velocity from the position difference. Thus, we utilize the planar positions, directional angle, and velocity as sensor observations for the Kalman smoother. More precisely, $z_{S}$ , $H_{S}$ , and $R_{S}$ are expressed as follows:

(22) $$\theta_{obs,S}\left(t\right)=atan2(y_{obs,S}\left(t+\frac{N_{S}∆t_{obs,S}}{2}\right)-y_{obs,S}\left(t-\frac{N_{S}∆t_{obs,S}}{2}\right),\;x_{obs,S}\left(t+\frac{N_{S}∆t_{obs,S}}{2}\right)-x_{obs,S}\left(t-\frac{N_{S}∆t_{obs,S}}{2}\right))$$

(23) $$v_{obs,S}\left(t\right)=\frac{\sqrt{{\left[x_{obs,S}\left(t+\frac{N_{S}∆t_{obs,S}}{2}\right)-x_{obs,S}\left(t-\frac{N_{S}∆t_{obs,S}}{2}\right)\right]}^{2}+{\left[y_{obs,S}\left(t+\frac{N_{S}∆t_{obs,S}}{2}\right)-y_{obs,S}\left(t-\frac{N_{S}∆t_{obs,S}}{2}\right)\right]}^{2}}}{N_{S}∆t_{obs,S}}$$

(24) $$z_{S}={\left(\begin{array}{cccc} x_{obs,S} & y_{obs,S} & \theta_{obs,S} & v_{obs,S} \end{array}\right)}^{T}$$

(25) $$H_{S}=\left(\begin{array}{cccccc} 1 & 0 & 0 & 0 & 0 & 0\\ 0 & 1 & 0 & 0 & 0 & 0\\ 0 & 0 & 1 & 0 & 0 & 0\\ 0 & 0 & 0 & 1 & 0 & 0 \end{array}\right)$$

(26) $$R_{S}=\left(\begin{array}{cccc} \sigma_{pos,S}^{2} & 0 & 0 & 0\\ 0 & \sigma_{pos,S}^{2} & 0 & 0\\ 0 & 0 & \sigma_{\theta ,S}^{2} & 0\\ 0 & 0 & 0 & \sigma_{v,S}^{2} \end{array}\right)$$

where $x_{obs,S}$ and $y_{obs,S}$ denote the positions, $\theta_{obs,S}$ denotes the directional angle, $v_{obs,S}$ denotes the velocity, $∆t_{obs,S}$ denotes the observation interval of the sensor, and $N_{S}$ denotes the number of time steps for stabilizing the calculation of the directional angle and the velocity. Here, because the directional angle and velocity are derived from the position difference, $\sigma_{\theta ,S}$ and $\sigma_{v,S}$ depend on $N_{S}$ , the accuracy of the position observation, and the cyclists’ actual velocity. Thus, these parameters are determined in Section 4.2.

#### 3.3.3. Clustering of Smoothed Data

Similarly to the variation in movement characteristics depending on the location, movement characteristics also vary among individuals. In the assumption of this study, individual optimization of movement characteristic parameters is impossible because the target cyclists who do not provide movement data are different from the cyclists who provide their movement data. However, if a cyclist observed by roadside sensors can be classified in the group whose characteristics are similar to those of the observed cyclist, the proposed system can select relatively adequate parameters. Thus, we cluster the smoothed data that have similar movement characteristics and derive the LDSI of each cluster.

Considering the above discussion, we designed a clustering method. As mentioned earlier, we first spatially resample the time-series smoothed data to the data linked with waypoints that have 1 m intervals between each other. Then, we cluster the resampled estimated data into multiple clusters. Because the movement data estimated by the Kalman smoother contain the uncertainty of each variable, consideration of the uncertainty is necessary for selecting the clustering method. Thus, we utilize the Wasserstein distance [34] as the metric between multiple data for clustering. More precisely, we select the 2-Wasserstein distance. The 2-Wasserstein distance between multivariate normal distributions $P_{W}$ and $Q_{W}$ , whose means are $\mu_{P\_W}$ and $\mu_{Q\_W}$ , and covariance matrices are $\Sigma_{P\_W}$ and $\Sigma_{Q\_W}$ , respectively, is expressed as follows [34]:

(27) $$D_{W_{2}}\left(P_{W},\;Q_{W}\right)=\sqrt{{\left|\mu_{P\_W}-\mu_{Q\_W}\right|}^{2}+trace\left(\Sigma_{P\_W}\right)+trace\left(\Sigma_{Q\_W}\right)-2trace\left[{\left(\sqrt{\Sigma_{P\_W}}\Sigma_{Q\_W}\sqrt{\Sigma_{P\_W}}\right)}^{\frac{1}{2}}\right]}$$

In this study, we calculate the 2-Wasserstein distance of the distribution $P_{W}$ and $Q_{W}$ of estimated movement state variables on each waypoint between the different data. Here, the above one indicates only the distance on the same waypoint of two different data. Thus, we then calculate the average 2-Wasserstein distance for all waypoints between each pair of data, and use the average distance for the clustering. As for the clustering algorithm, we use hierarchical clustering. In addition, from the viewpoint of the calculation method for the distance between clusters, we use unweighted average linkage clustering.

To calculate the Wasserstein distance, we must consider the variables used for clustering. From the viewpoint of collision prevention applications, it is important to estimate the offset, which is the position of the cyclist along the road. At this point, the velocity is directly useful for estimating the offset. In contrast, because the directional angle and yawing rate of cyclists differ little on a straight road, we omitted these variables for the clustering criteria. Additionally, because we confirmed that acceleration did not work well for clustering in the preliminary analysis, we also omitted acceleration. Consequently, we calculate the Wasserstein distance based only on the estimated velocity.

After the calculation of the distance between each pair of data, we divide all the data into a predetermined number of clusters based on the dendrogram. In each cluster, we prepare LDSI by calculating the mean values and standard deviations of the estimated variables at each waypoint.

### 3.4. Movement States Estimation Using LDSI

#### 3.4.1. Real-Time Movement States Estimation Using Kalman Filter

In the real-time estimation of the movement state using the EKF, we define the state variables $x_{F}$ , the estimation covariance matrix $P_{F}$ , and the control input $u_{F}$ as follows:

(28) $$x_{F}={\left(\begin{array}{cccc} x & y & \theta & v \end{array}\right)}^{T}$$

(29) $$P_{F}=\left(\begin{array}{cccc} \sigma_{x}^{2} & \sigma_{xy} & \sigma_{x\theta } & \sigma_{xv}\\ \sigma_{xy} & \sigma_{y}^{2} & \sigma_{y\theta } & \sigma_{yv}\\ \sigma_{x\theta } & \sigma_{y\theta } & \sigma_{\theta }^{2} & \sigma_{\theta v}\\ \sigma_{xv} & \sigma_{yv} & \sigma_{\theta v} & \sigma_{v}^{2} \end{array}\right)$$

(30) $$u_{F}={\left(\begin{array}{cc} \omega & a \end{array}\right)}^{T}$$

The equations for the EKF are as follows:

(31) $$\begin{array}{c} x_{F}\left(t_{k}\right)=f_{F}(x_{F}\left(t_{k-1}\right),u_{F}\left(t_{k-1}\right)) \end{array}$$

(32) $$z_{F}\left(t_{k}\right)=h_{F}(x_{F}\left(t_{k}\right))$$

where $f_{F}$ denotes the state transition function, and $h_{F}$ denotes the observation function. The prediction part is expressed as follows:

(33) $${\hat{x}}_{F}\left(t_{k}|t_{k-1}\right)=f_{F}\left({\hat{x}}_{F}\left(t_{k-1}|t_{k-1}\right),u_{F}\left(t_{k-1}\right)\right)$$

(34) $$P_{F}\left(t_{k}|t_{k-1}\right)=F_{F}\left(t_{k-1}\right)P_{F}\left(t_{k-1}|t_{k-1}\right)F_{F}^{T}\left(t_{k-1}\right)+B_{F}\left(t_{k-1}\right)Q_{F}\left(t_{k-1}\right)B_{F}^{T}\left(t_{k-1}\right)$$

where ${\hat{x}}_{F}$ denotes the filtered state, $F_{F}$ denotes the matrix of the partial derivatives of $f_{F}$ by $x_{F}$ , $B_{F}$ denotes the matrix of the partial derivatives of $f_{F}$ by $u_{F}$ , and $Q_{F}$ denotes the process noise matrix. The update part is expressed as follows:

(35) $$S_{F}\left(t_{k}|t_{k-1}\right)=H_{F}P_{F}\left(t_{k}|t_{k-1}\right)H_{F}^{T}+R_{F}\left(t_{k}\right)$$

(36) $$K_{F}\left(t_{k}\right)=P_{F}\left(t_{k}|t_{k-1}\right)H_{F}^{T}S_{F}^{-1}\left(t_{k}|t_{k-1}\right)$$

(37) $${\hat{x}}_{F}\left(t_{k}|t_{k}\right)={\hat{x}}_{F}\left(t_{k}|t_{k-1}\right)+K_{F}\left(t_{k}\right)\left[z_{F}\left(t_{k}\right)-h_{F}\left({\hat{x}}_{F}\left(t_{k}|t_{k-1}\right)\right)\right]$$

(38) $$P_{F}\left(t_{k}|t_{k}\right)=\left[I-K_{F}\left(t_{k}\right)H_{F}\right]P_{F}\left(t_{k}|t_{k-1}\right){\left[I-K_{F}\left(t_{k}\right)H_{F}\right]}^{T}+K_{F}\left(t_{k}\right)R_{F}\left(t_{k}\right)K_{F}^{T}\left(t_{k}\right)$$

where $S_{F}$ denotes the system uncertainty matrix, $H_{F}$ denotes the matrix of the partial derivatives of $h_{F}$ by $x_{F}$ , $R_{F}$ denotes the measurement noise matrix, and $K_{F}$ denotes the Kalman gain.

Unlike the Kalman smoother in the preprocessing part, we assume that only the uncertainty of the control input affects the estimation covariance matrix $P_{F}$ in real-time movement state estimation using the Kalman filter. Thus, $f_{F}$ is expressed as follows:

(39) $$f_{F}\left(x_{F}\left(t_{k-1}\right),u_{F}\left(t_{k-1}\right)\right)=\left(\begin{array}{c} x\left(t_{k-1}\right)+v\left(t_{k-1}\right)∆t\cos\theta \left(t_{k-1}\right)+\frac{1}{2}a\left(t_{k-1}\right)∆t^{2}\cos\theta \left(t_{k-1}\right)\\ y\left(t_{k-1}\right)+v\left(t_{k-1}\right)∆t\sin\theta \left(t_{k-1}\right)+\frac{1}{2}a\left(t_{k-1}\right)∆t^{2}\sin\theta \left(t_{k-1}\right)\\ \theta \left(t_{k-1}\right)+\omega \left(t_{k-1}\right)∆t\\ v\left(t_{k-1}\right)+a\left(t_{k-1}\right)∆t \end{array}\right)$$

where ∆t denotes the time step between the predictions. Moreover, the process noise matrix $Q_{F}$ is as follows:

(40) $$Q_{F}\left(t_{k-1}\right)=\left(\begin{array}{cc} \sigma_{\omega }^{2}\left(t_{k-1}\right) & 0\\ 0 & \sigma_{a}^{2}\left(t_{k-1}\right) \end{array}\right)$$

From the above discussion, $F_{F}$ and $B_{F}$ are derived as follows:

(41) $$\begin{array}{cc} F_{F}\left(t_{k-1}\right) & =\frac{\partial f_{F}\left(x_{F},u_{F}\right)}{\partial x_{F}}{|}_{x_{F}=x_{F}\left(t_{k-1}\right),u_{F}=u_{F}\left(t_{k-1}\right)}\\ & \;\;\;=\left(\begin{array}{cccc} 1 & 0 & -v\left(t_{k-1}\right)∆t\sin\theta \left(t_{k-1}\right)-\frac{1}{2}a\left(t_{k-1}\right)∆t^{2}\sin\theta \left(t_{k-1}\right) & ∆t\cos\theta \left(t_{k-1}\right)\\ 0 & 1 & v\left(t_{k-1}\right)∆t\cos\theta \left(t_{k-1}\right)+\frac{1}{2}a\left(t_{k-1}\right)∆t^{2}\cos\theta \left(t_{k-1}\right) & ∆t\sin\theta \left(t_{k-1}\right)\\ 0 & 0 & 1 & 0\\ 0 & 0 & 0 & 1 \end{array}\right) \end{array}$$

(42) $$B_{F}\left(t_{k-1}\right)=\frac{\partial f_{F}\left(x_{F},u_{F}\right)}{\partial u_{F}}{|}_{x_{F}=x_{F}\left(t_{k-1}\right)}=\left(\begin{array}{cc} 0 & \frac{1}{2}∆t^{2}\cos\theta \left(t_{k-1}\right)\\ 0 & \frac{1}{2}∆t^{2}\sin\theta \left(t_{k-1}\right)\\ ∆t & 0\\ 0 & ∆t \end{array}\right)$$

Here, we first assume that the control input $u_{F}$ is **0**, and the process noise $Q_{F}$ has the same value in the preprocessing part when there is no available information. When the virtual observation is used, virtual control input $u_{v}$ and corresponding process noise $Q_{v}$ based on LDSI are assigned to $u_{F}$ and $Q_{F}$ , respectively. The specific calculation method is described in Section 3.4.3.

The remaining undetermined variables are $z_{F}$ , $H_{F}$ , and $R_{F}$ . Because we utilize the virtual observation to compensate for the lack of actual sensor observation, the virtual observation is utilized only when actual sensor observation is not available. Thus, if actual sensor observation is available, corresponding values of actual sensors are assigned to $z_{F}$ , $H_{F}$ , and $R_{F}$ . Otherwise, virtual observation $z_{v}$ , $H_{v}$ , and $R_{v}$ based on LDSI are assigned to $z_{F}$ , $H_{F}$ , and $R_{F}$ . Additionally, because these variables depend on the sensor characteristics, the variables of the actual sensor observation and virtual observation are different from each other. Thus, we determine the variables of the actual sensor observation and the virtual observation in Section 3.4.2 and Section 3.4.3, respectively.

#### 3.4.2. Implementation of Actual Sensor Observation

In existing studies, various types of sensors, such as cameras, LiDARs, and radars, have been utilized as roadside sensors. Because not all types of sensors can directly observe directional angle and velocity, we assume to calculate the directional angle and velocity from the position difference when the sensors do not provide such information. Thus, similar to the observation in the preprocessing part, we utilize the position, directional angle, and velocity as sensor observations. More precisely, $z_{F}$ , $H_{F}$ , and $R_{F}$ . are expressed as follows:

(43) $$\theta_{obs,F}\left(t\right)=atan2\left(y_{obs,F}\left(t\right)-y_{obs,F}\left(t-N_{F}∆t_{obs,F}\right),x_{obs,F}\left(t\right)-x_{obs,F}\left(t-N_{F}∆t_{obs,F}\right)\right)$$

(44) $$v_{obs,F}\left(t\right)=\frac{\sqrt{{\left[x_{obs,F}\left(t\right)-x_{obs,F}\left(t-N_{F}∆t_{obs,F}\right)\right]}^{2}+{\left[y_{obs,F}\left(t\right)-y_{obs,F}\left(t-N_{F}∆t_{obs,F}\right)\right]}^{2}}}{N_{F}∆t_{obs,F}}$$

(45) $$z_{F}={\left(\begin{array}{cccc} x_{obs,F} & y_{obs,F} & \theta_{obs,F} & v_{obs,F} \end{array}\right)}^{T}$$

(46) $$H_{F}=\left(\begin{array}{cccc} 1 & 0 & 0 & 0\\ 0 & 1 & 0 & 0\\ 0 & 0 & 1 & 0\\ 0 & 0 & 0 & 1 \end{array}\right)$$

(47) $$R_{F}=\left(\begin{array}{cccc} \sigma_{pos,F}^{2} & 0 & 0 & 0\\ 0 & \sigma_{pos,F}^{2} & 0 & 0\\ 0 & 0 & \sigma_{\theta ,F}^{2} & 0\\ 0 & 0 & 0 & \sigma_{v,F}^{2} \end{array}\right)$$

where $∆t_{obs,F}$ denotes the observation interval of a sensor, and $N_{F}$ denotes the number of time steps for stabilizing the calculation of the directional angle and velocity. Here, $∆t_{obs,F}$ and $N_{F}$ are different from the corresponding values in the preprocessing part. On the contrary, as discussed in Section 3.3.2, $\sigma_{\theta ,F}$ and $\sigma_{v,F}$ depend on $N_{F}$ , the accuracy of the position observation, and the cyclists’ actual velocity. Thus, these parameters are determined in Section 4.3.

#### 3.4.3. Implementation of Virtual Control Input and Virtual Observation

**Figure 8:**
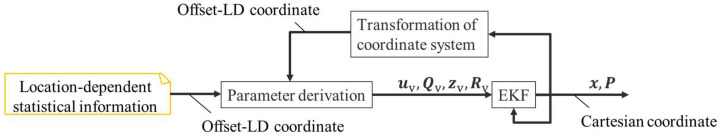
Pipeline of movement estimation using LDSI.

Because both the virtual observation and virtual control input are calculated from the LDSI that is associated with waypoints, the estimated current position is required for referring to the LDSI. However, the coordinate system of the EKF is different from that of the LDSI; the EKF is based on the Cartesian coordinate system, and the LDSI is based on the Offset-LD coordinate system, as shown in Figure 6. Thus, the transformation of coordinate systems is necessary. Figure 8 shows the pipeline of movement estimation related to the transformation of the coordinate systems. The transformation of the coordinate system is expressed as follows:

(48) $$M_{\psi }=\left(\begin{array}{cc} \cos\psi & -\sin\psi \\ \sin\psi & \cos\psi \end{array}\right)$$

(49) $$\left(\begin{array}{c} \hat{o}\\ \hat{l} \end{array}\right)=M_{\psi }^{-1}\left(\begin{array}{c} \hat{x}-x_{C}\\ \hat{y}-y_{C} \end{array}\right)+\left(\begin{array}{c} o_{C}\\ l_{C} \end{array}\right)$$

where ψ denotes the angle from the x-axis to the offset-axis with counterclockwise rotation as positive, $M_{\psi }$ denotes the rotation matrix, $x_{C}$ and $y_{C}$ denote the planar positions of the closest waypoint from the current position, $\hat{o}$ and $\hat{l}$ denote the estimated offset and LD in the Offset-LD coordinate system, and $o_{C}$ and $l_{C}$ denote the offset and LD of the closest waypoint from the current position, respectively. The transformation of the coordinate system for the estimation covariance matrix is expressed as follows:

(50) $$\left(\begin{array}{cc} \sigma_{o}^{2} & \sigma_{ol}\\ \sigma_{ol} & \sigma_{l}^{2} \end{array}\right)=M_{\psi }^{-1}\left(\begin{array}{cc} \sigma_{x}^{2} & \sigma_{xy}\\ \sigma_{xy} & \sigma_{y}^{2} \end{array}\right)M_{\psi }$$

where $\sigma_{o}$ and $\sigma_{l}$ denote the estimation uncertainties of the offset and LD positions, respectively, and $\sigma_{ol}$ denotes the covariance of the offset and LD positions.

**Figure 9:**
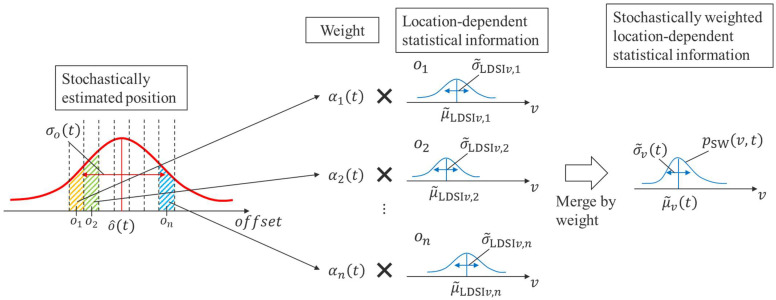
Conceptual schematic of the SWLDSI calculation. The red line in the left graph indicates stochastic position distribution of the target cyclist along offset axis.

In addition, we must consider the uncertainty of the estimated current position. Thus, we calculate the probability of the current position of the cyclist for each waypoint based on the estimated movement state. Based on the probability, we calculate stochastically weighted location-dependent statistical information (SWLDSI). Figure 9 shows a conceptual schematic of this process.

First, the existence probability $\alpha_{i}$ of a cyclist for the *i*-th waypoint at time step t is calculated from the probability distribution function of the position of a cyclist along the road. To be more detailed, $\alpha_{i}\left(t\right)$ is approximately calculated as follows:

(51) $$\alpha_{i}\left(t\right)≅\int_{o_{i}-0.5}^{o_{i}+0.5}\frac{1}{\sqrt{2\pi \sigma_{o}^{2}\left(t\right)}}\exp\left\{-\frac{{\left[o-\hat{o}\left(t\right)\right]}^{2}}{2\sigma_{o}^{2}\left(t\right)}\right\}do$$

where $o_{i}$ denotes offset of the *i*-th waypoint, $\hat{o}\left(t\right)$ denotes the estimated offset at time step t along the road, and $\sigma_{o}\left(t\right)$ denotes its uncertainty.

Next, we need to design a method to calculate SWLDSI based on the existence probability for each waypoint. The LDSI is linked with each waypoint and consists of the mean values and standard deviations of the movement state. Thus, we calculate the SWLDSI by weighting the LDSI with the above existence probability $\alpha_{i}$ . To be more detailed, we assume weighted average of probability distribution of LDSI related to velocity v at each way point as probability distribution $p_{SW}\left(v,\;t\right)$ of SWLDSI, as shown in Equation (52).

(52) $$p_{SW}\left(v,t\right)≔\sum_{i=1}^{n}\alpha_{i}\left(t\right)\frac{1}{\sqrt{2\pi {\tilde{\sigma }}_{LDSIv,i}^{2}}}\exp\left[-\frac{{\left(v-{\tilde{\mu }}_{LDSIv,i}\right)}^{2}}{2{\tilde{\sigma }}_{LDSIv,i}^{2}}\right]$$

where n denotes the number of waypoint, and ${\tilde{\mu }}_{LDSIv,i}$ and ${\tilde{\sigma }}_{LDSIv,i}$ denote the LDSI at the *i*-th waypoint. Here, we assume probability distribution of LDSI at each way point as the normal distribution. In practice, the mean ${\tilde{\mu }}_{v}\left(t\right)$ and the standard deviation ${\tilde{\sigma }}_{v}\left(t\right)$ of the SWLDSI at time step t are calculated as follows:

(53) $${\tilde{\mu }}_{v}\left(t\right)=\sum_{i=1}^{n}\alpha_{i}\left(t\right){\tilde{\mu }}_{LDSIv,i}$$

(54) $${\tilde{\sigma }}_{v}^{2}\left(t\right)=\sum_{i=1}^{n}\alpha_{i}\left(t\right){\tilde{\sigma }}_{LDSIv,i}^{2}+\sum_{i=1}^{n}\alpha_{i}\left(t\right){\tilde{\mu }}_{LDSIv,i}^{2}-{\left[\sum_{i=1}^{n}\alpha_{i}\left(t\right){\tilde{\mu }}_{LDSIv,i}\right]}^{2}$$

The detailed derivations are given in the Appendix A. From Equation (53), ${\tilde{\mu }}_{v}$ is derived by the weighted average of ${\tilde{\mu }}_{LDSIv,i}$ . Additionally, from Equation (54), ${\tilde{\sigma }}_{v}^{2}$ is derived by the summation of the weighted average of ${\tilde{\sigma }}_{LDSIv,i}^{2}$ and the variance of ${\tilde{\mu }}_{LDSIv,i}$ . Similarly to velocity, the SWLDSI of the directional angle, yawing rate, and acceleration are calculated as follows:

(55) $${\tilde{\mu }}_{\theta }\left(t\right)=\sum_{i=1}^{n}\alpha_{i}\left(t\right){\tilde{\mu }}_{LDSI\theta ,i}$$

(56) $${\tilde{\sigma }}_{\theta }^{2}\left(t\right)=\sum_{i=1}^{n}\alpha_{i}\left(t\right){\tilde{\sigma }}_{LDSI\theta ,i}^{2}+\sum_{i=1}^{n}\alpha_{i}\left(t\right){\tilde{\mu }}_{LDSI\theta ,i}^{2}-{\left[\sum_{i=1}^{n}\alpha_{i}\left(t\right){\tilde{\mu }}_{LDSI\theta ,i}\right]}^{2}$$

(57) $${\tilde{\mu }}_{\omega }\left(t\right)=\sum_{i=1}^{n}\alpha_{i}\left(t\right){\tilde{\mu }}_{LDSI\omega ,i}$$

(58) $${\tilde{\sigma }}_{\omega }^{2}\left(t\right)=\sum_{i=1}^{n}\alpha_{i}\left(t\right){\tilde{\sigma }}_{LDSI\omega ,i}^{2}+\sum_{i=1}^{n}\alpha_{i}\left(t\right){\tilde{\mu }}_{LDSI\omega ,i}^{2}-{\left[\sum_{i=1}^{n}\alpha_{i}\left(t\right){\tilde{\mu }}_{LDSI\omega ,i}\right]}^{2}$$

(59) $${\tilde{\mu }}_{a}\left(t\right)=\sum_{i=1}^{n}\alpha_{i}\left(t\right){\tilde{\mu }}_{LDSIa,i}$$

(60) $${\tilde{\sigma }}_{a}^{2}\left(t\right)=\sum_{i=1}^{n}\alpha_{i}\left(t\right){\tilde{\sigma }}_{LDSIa,i}^{2}+\sum_{i=1}^{n}\alpha_{i}\left(t\right){\tilde{\mu }}_{LDSIa,i}^{2}-{\left[\sum_{i=1}^{n}\alpha_{i}\left(t\right){\tilde{\mu }}_{LDSIa,i}\right]}^{2}$$

Owing to the above SWLDSI, even if the current estimated position contains large uncertainty and is located around the area where LDSI changes greatly, we can adequately reflect the uncertainty of the estimated movement state.

Additionally, to stably estimate moving states that contain relatively outliers among the clusters, we introduce safety factors ${sf}_{Q}$ and ${sf}_{R}$ to the process noise and observation noise, respectively. SWLDSI with safety factors is expressed as follows:

(61) $${\tilde{\sigma }}_{\theta }^{\prime }\left(t_{k}\right)=\left(1+{sf}_{R}\right){\tilde{\sigma }}_{\theta }\left(t_{k}\right)$$

(62) $${\tilde{\sigma }}_{v}^{\prime }\left(t_{k}\right)=\left(1+{sf}_{R}\right){\tilde{\sigma }}_{v}\left(t_{k}\right)$$

(63) $${\tilde{\sigma }}_{\omega }^{\prime }\left(t_{k}\right)=\left(1+{sf}_{Q}\right){\tilde{\sigma }}_{\omega }\left(t_{k}\right)$$

(64) $${\tilde{\sigma }}_{a}^{\prime }\left(t_{k}\right)=\left(1+{sf}_{Q}\right){\tilde{\sigma }}_{a}\left(t_{k}\right)$$

${sf}_{Q}$ and ${sf}_{R}$ are not the analyzed parameters but the design parameters, and we assigned 0.3 to these parameters in this study.

**Figure 10:**
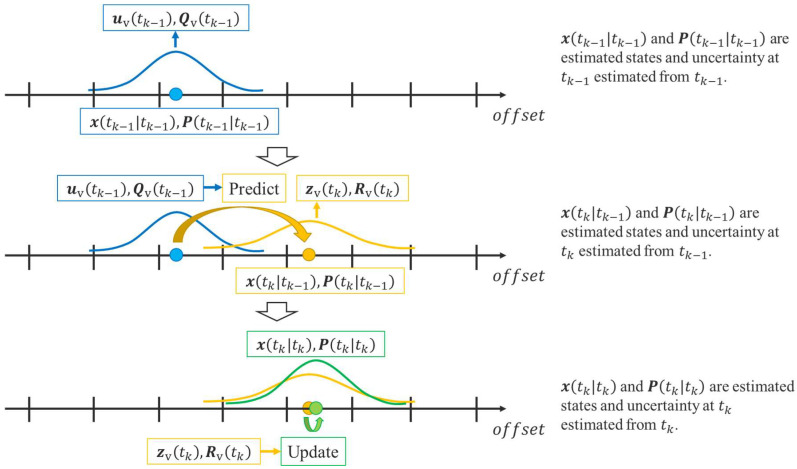
Conceptual schematic of the calculation process for virtual observation and virtual control input. The colors in the figure indicate the time step of estimated variables. Blue indicates the estimated variables at $t_{k-1}$ estimated from $t_{k-1}$ . Yellow indicates the estimated variables at $t_{k}$ estimated from $t_{k-1}$ . Green indicates the estimated variables at $t_{k}$ estimated from $t_{k}$ .

SWLDSI is utilized for the calculation of the virtual control input $u_{v}$ , process noise $Q_{v}$ , virtual observation $z_{v}$ , and observation noise $R_{v}$ , respectively. Figure 10 shows a conceptual schematic of the calculation process. The virtual control input $u_{v}$ and process noise $Q_{v}$ at time step $t_{k-1}$ are calculated from SWLDSI based on the results of the EKF update at time step $t_{k-1}$ , and used for the prediction of the EKF from time step $t_{k-1}$ . The virtual observation $z_{v}$ and observation noise $R_{v}$ are calculated from SWLDSI based on the latest results of the EKF prediction. The movement state at time step $t_{k}$ are updated using the above virtual observations and virtual control inputs.

As for the virtual control input $u_{v}$ , virtual observation $z_{v}$ , and process noise $Q_{v}$ , SWLDSI is directly applied to EKF. In other words, each value for EKF is expressed as follows:

(65) $$u_{v}\left(t_{k-1}\right)={\left(\begin{array}{cc} {\tilde{\mu }}_{\omega }\left(t_{k-1}\right) & {\tilde{\mu }}_{a}\left(t_{k-1}\right) \end{array}\right)}^{T}$$

(66) $$Q_{v}\left(t_{k-1}\right)=\left(\begin{array}{cc} {{\tilde{\sigma }}_{\omega }^{\prime }}^{2}\left(t_{k-1}\right) & 0\\ 0 & {{\tilde{\sigma }}_{a}^{\prime }}^{2}\left(t_{k-1}\right) \end{array}\right)$$

(67) $$z_{v}\left(t_{k}\right)={\left(\begin{array}{cc} {\tilde{\mu }}_{\theta }\left(t_{k}\right) & {\tilde{\mu }}_{v}\left(t_{k}\right) \end{array}\right)}^{T}$$

(68) $$H_{v}=\left(\begin{array}{cccc} 0 & 0 & 1 & 0\\ 0 & 0 & 0 & 1 \end{array}\right)$$

As for the observation noise $R_{v}$ , we need to consider the time-series effect of $R_{v}$ . In general, if the EKF process is repeated, the element of the estimation covariance matrix P becomes smaller than the corresponding value of the observation noise $R_{v}$ . Thus, to avoid making the estimation covariance matrix P excessively small, the observation noise $R_{v}$ must be adjusted so that the estimation covariance matrix P via repeated virtual observations converges to SWLDSI, similar to our previous study [7]. Here, the uncertainty of the directional angle and the velocity are not affected by the position uncertainty. Thus we calculate the modified $R_{v}$ by solving the algebraic Riccati Equations [35] for the directional angle and velocity. $R_{v}$ is expressed as follows:

(69) $$\sigma_{\theta \_v}\left(t_{k}\right)=\frac{\sqrt{{{\tilde{\sigma }}_{\theta }^{\prime }}^{4}\left(t_{k}\right)+{{\tilde{\sigma }}_{\omega }^{\prime }}^{2}\left(t_{k-1}\right)∆t^{2}{{\tilde{\sigma }}_{\theta }^{\prime }}^{2}\left(t_{k}\right)}}{{\tilde{\sigma }}_{\omega }^{\prime }\left(t_{k-1}\right)∆t}$$

(70) $$\sigma_{v\_v}\left(t_{k}\right)=\frac{\sqrt{{{\tilde{\sigma }}_{v}^{\prime }}^{4}\left(t_{k}\right)+{{\tilde{\sigma }}_{a}^{\prime }}^{2}\left(t_{k-1}\right)∆t^{2}{{\tilde{\sigma }}_{v}^{\prime }}^{2}\left(t_{k}\right)}}{{\tilde{\sigma }}_{a}^{\prime }\left(t_{k-1}\right)∆t}$$

(71) $$R_{v}\left(t_{k}\right)=\left(\begin{array}{cc} \sigma_{\theta \_v}^{2}\left(t_{k}\right) & 0\\ 0 & \sigma_{v\_v}^{2}\left(t_{k}\right) \end{array}\right)$$

where ∆t denotes the observation cycle time. Here, we need to design an adequate cycle time for the prediction of the EKF, which is also equivalent to the observation cycle. Because the values of the virtual control input $u_{v}$ and process noise $Q_{v}$ are estimated from the accumulated GNSS data, these values are based on the assumption of constant acceleration and constant yawing motion within the observation cycle time of GNSS. Thus, it is desirable to match the cycle time of the EKF prediction, which uses virtual control input $u_{v}$ and process noise $Q_{v}$ , to the GNSS observation cycle time. Based on the above consideration, we determine the cycle time for virtual observation and virtual control input as 1.0 s, which is equivalent to the cycle time of GNSS used in the experiment. Using variables derived by the above discussion, we estimate the cyclists’ movement states with the virtual control input and virtual observation when actual sensor observation is not available, as mentioned in Section 3.4.1.

## 4. Experiment for Preparing LDSI

### 4.1. Experimental Setup

**Figure 11:**
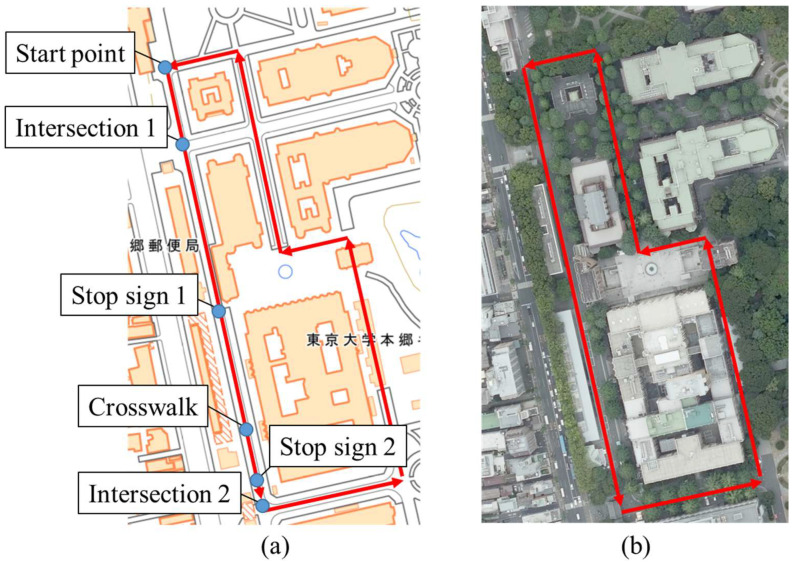
(**a**) Map of the experiment site (created by editing the digital map [36]). (**b**) Aerial photo of the experiment site (created by editing the aerial photograph [37]). The Chinese characters on the left side of (**a**) indicate a “post office”, and the Chinese characters on the right side of (**a**) indicate the “Hongo campus of the University of Tokyo”.

We conducted an experiment to accumulate GNSS data in the Hongo campus of the University of Tokyo. Although the roads in the campus of the university are not actual community roads, the traffic characteristics of them are similar to those of community roads; pedestrians and cyclists move, and the visibility around nonsignalized intersections is bad. Thus, we considered the road in the university campus as pseudo community roads, and we conducted the experiment in the university campus. The study was conducted in accordance with the Declaration of Helsinki, and the protocol for this study was approved by Research Ethics Committee, School of Engineering, The University of Tokyo (KE24-52) on 21 October 2024. We recruited 10 experimental participants with ages ranged from twenties to thirties. We obtained written informed consent from the participants. The participants rode on a bicycle and moved along the experimental course three times. Figure 11 shows the appearances of the experimental course [36,37]. The course length was approximately 840 m. During the experiment, a smartphone was attached to the bicycle, and it accumulated GNSS data. Although we recorded 30 data in total, one participant took the wrong course once. Thus, we finally obtained a total of 29 valid data.

### 4.2. Parameters Related to GNSS Observation for Kalman Smoother

The observation frequency of GNSS was 1 Hz. Because we used a typical smartphone to obtain GNSS data, the observation accuracy of the GNSS was not very high. Thus, we assumed that $\sigma_{pos,\;S}$ for the GNSS was 4.25 m. In addition, we calculated the directional angle and velocity via Equations (22) and (23). As for the $\sigma_{\theta ,\;S}$ and $\sigma_{v,\;S}$ , we determined the values from the preliminary numerical simulation. More precisely, we added noise, whose average was zero and the standard deviation was $\sigma_{pos,\;S}$ , to the ground truth value of the position on the assumption that the average velocity of cyclists was 4.2 m/s. Then, we calculated the distribution of θ and v , and determined the $N_{S}$ , $\sigma_{\theta ,\;S}$ , and $\sigma_{v,\;S}$ as follows:

(72) $$N_{S}=2$$

(73) $$\sigma_{\theta ,S}=0.88\;rad$$

(74) $$\sigma_{v,S}=2.8\;m/s$$

As a result, $R_{S}$ was expressed as follows:

(75) $$R_{S}=\left(\begin{array}{cccc} \sigma_{pos,S}^{2} & 0 & 0 & 0\\ 0 & \sigma_{pos,S}^{2} & 0 & 0\\ 0 & 0 & \sigma_{\theta ,S}^{2} & 0\\ 0 & 0 & 0 & \sigma_{v,S}^{2} \end{array}\right)=\left(\begin{array}{cccc} 4.25^{2} & 0 & 0 & 0\\ 0 & 4.25^{2} & 0 & 0\\ 0 & 0 & 0.88^{2} & 0\\ 0 & 0 & 0 & 2.8^{2} \end{array}\right)$$

### 4.3. Clustering Result

We analyzed the movement characteristics of the cyclists using the accumulated GNSS data. We focused on the movement data on a straight road from the start point to intersection 2, as shown in Figure 11.

**Figure 12:**
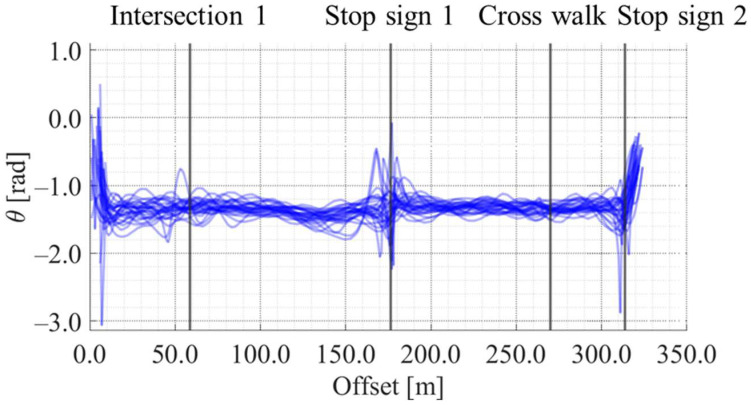
Accumulated directional angle data along offset axis.

**Figure 13:**
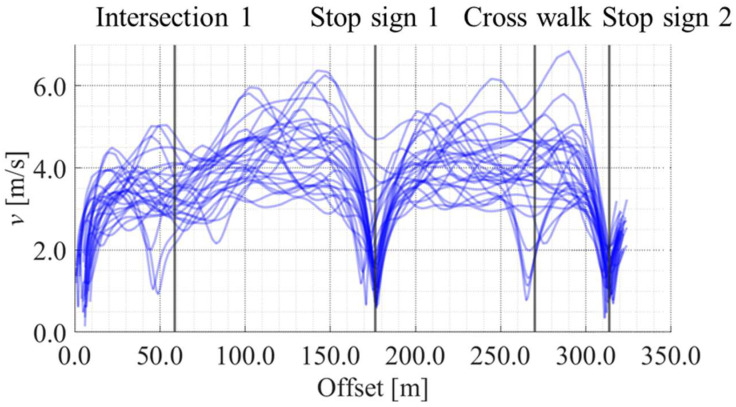
Accumulated velocity data along the offset axis.

**Figure 14:**
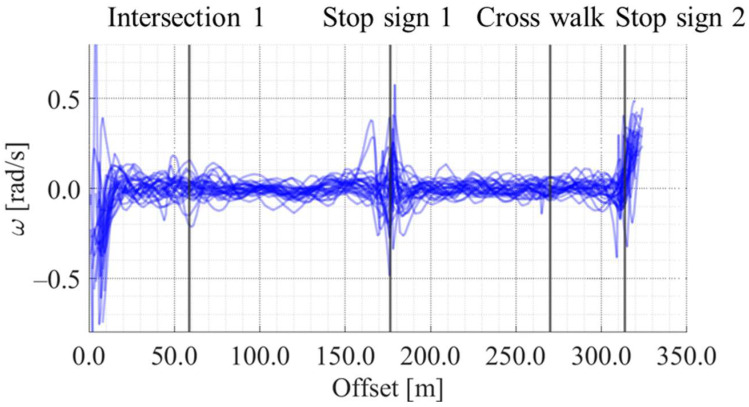
Accumulated yawing rate data along the offset axis.

**Figure 15:**
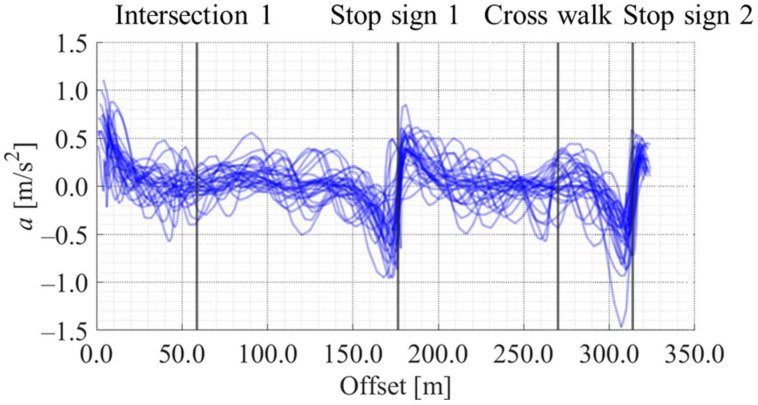
Accumulated acceleration data along the offset axis.

Figure 12, Figure 13, Figure 14 and Figure 15 show the directional angle, velocity, yawing rate, and acceleration, respectively, estimated by the Kalman smoother. The horizontal axis in each figure indicates the offset, which is the distance from the start point along the road. In addition, a directional angle of 0 rad indicates the East direction.

Figure 13 indicates that the estimated velocity in almost all trials decreased around stop signs 1 and 2, which corresponds to the areas whose offset are approximately 175 m and 315 m, respectively. Additionally, Figure 13 indicates that the estimated velocity in some trials decreased around intersection 1 and the crosswalk, which corresponds to the areas whose offset are approximately 60 m and 270 m, respectively. Such location-dependent movement characteristics were also confirmed for the other state variables. Thus, the LDSI can be calculated from the estimated values.

**Figure 16:**
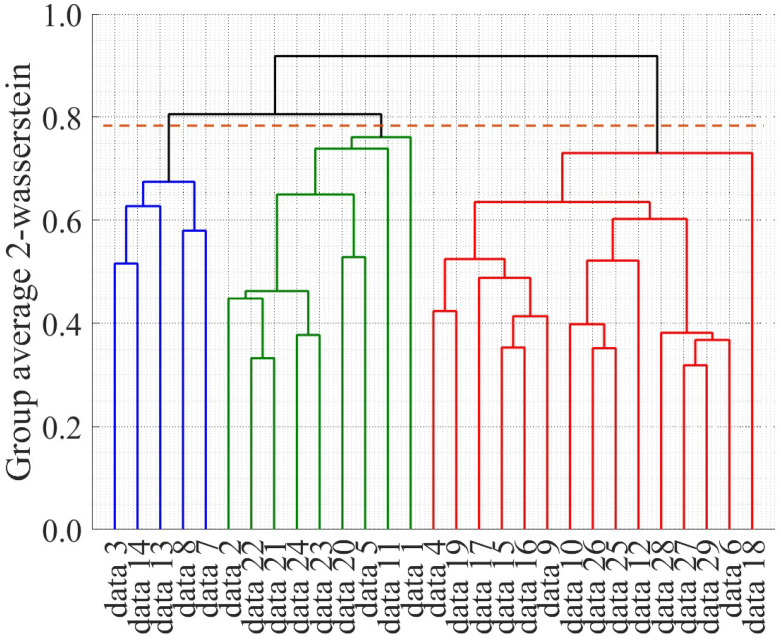
Dendrogram of clustering results. The blue lines, the green lines, and the red lines indicate the data in Cluster 1, data in Cluster 2, and the data in Cluster 3, respectively. The orange dashed line indicates the cut-off line.

**Figure 17:**
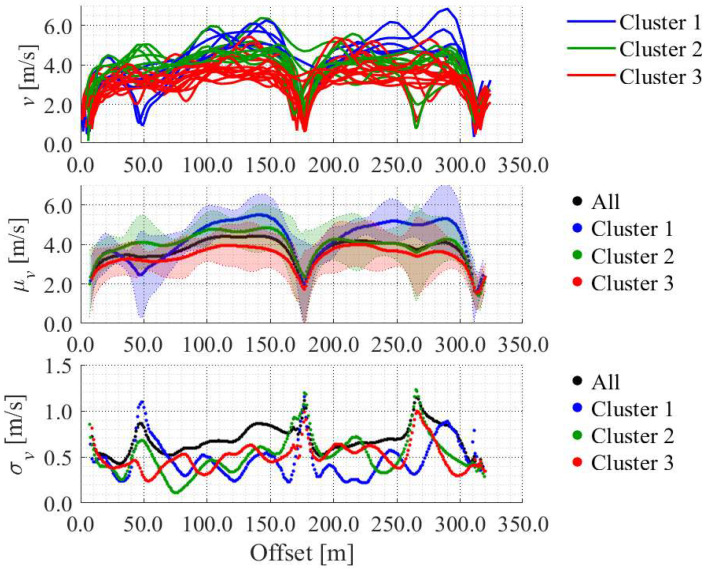
Velocity in each cluster.

Next, we clustered the accumulated data based on the estimated velocities. Figure 16 shows the dendrogram of the clustering results. As an initial step in the approach proposed in this study, we divided the obtained data into three groups based on the dendrogram. The top, middle, and bottom graphs in Figure 17 show the estimated velocity in each cluster, average velocity with 2 σ range in each cluster, and standard deviation of the velocity in each cluster, respectively. The data in Cluster 1 tended to show a large velocity change. The data in Cluster 2 tended to show high velocity. The data in Cluster 3 tended to show low velocity. Additionally, the standard deviation in each cluster was smaller than that of all the data without clustering. Therefore, the clustering process enables us to understand the movement characteristics in more detail.

**Figure 18:**
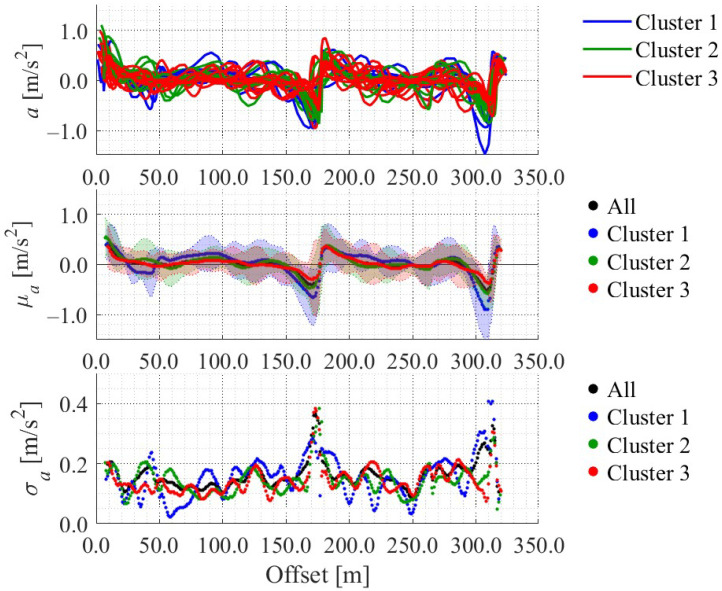
Acceleration in each cluster.

**Figure 19:**
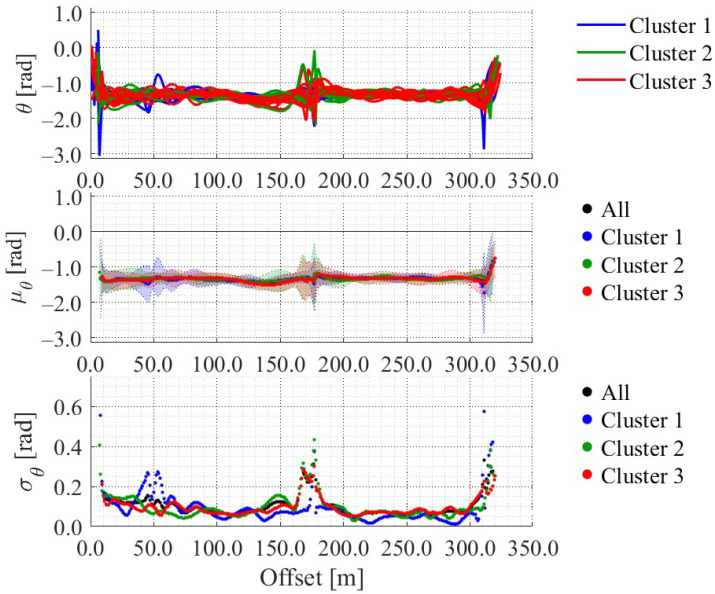
Directional angle in each cluster.

**Figure 20:**
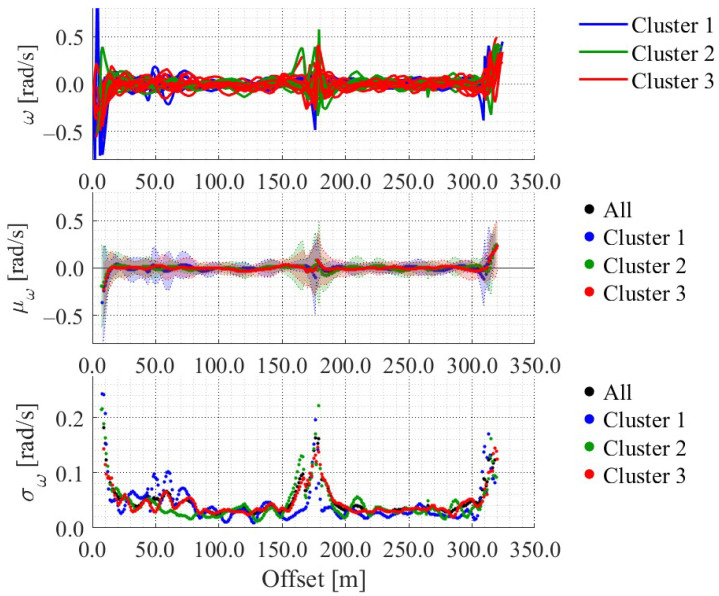
Yawing rate in each cluster.

Based on the above clustering, we also classified the directional angle data, yawing rate data, and acceleration data. Figure 18, Figure 19 and Figure 20 show the results. We calculated the LDSI related to the virtual observation and virtual control input based on the distribution of these variables, and utilized the LDSI for position estimation in the following section.

## 5. Simulation Experiment of Position Estimation Using LDSI

### 5.1. Data for Simulation Experiment

In this simulation experiment, we used the same data explained in Section 4. Thus, we used the values estimated by the Kalman smoother as the ground truth for position estimation. To be more precise, we linearly interpolated the estimated values, which were estimated using the Kalman smoother at 1 Hz, to values at 10 Hz, and used the interpolated values as the ground truth.

As shown in Figure 4, the observation of the cyclist by the roadside sensor at the first intersection enables our proposed system to continue estimating the position even after the cyclist moves out from the observation range of the roadside sensor. To confirm the effectiveness of this function, we set the observation range of the virtual roadside sensor, and utilized the ground truth values of the position as observation values by the roadside sensor. Additionally, although the classification function of the observed cyclist into a predetermined cluster corresponding to the LDSI was necessary to realize the proposed system, the main purpose of this study was to confirm the effectiveness of using LDSI. Thus, we assumed an ideal classification function and evaluated the effectiveness of the position estimation using LDSI.

### 5.2. Parameters of Roadside Sensor Observation

We assumed LiDAR as the roadside sensor for this simulation. Although LiDAR obtains point clouds with precise accuracy, the clustering process for detecting traffic participants produces positioning errors to a certain extent. Thus, we assumed the $\sigma_{pos,\;F}$ of LiDAR observation as 0.1 m. In addition, we set the observation range as a circular area with a radius of 10 m, and observation cycle as 10 Hz. As for the $\sigma_{\theta ,\;F}$ and $\sigma_{v,\;F}$ , we preliminarily determined the values from the numerical simulation, similar to the case in Section 4.2. More precisely, we added noise, whose average was zero and standard deviation was $\sigma_{pos,\;F}$ , to the ground truth value of the position on the assumption that the average velocity of cyclists was 4.2 m/s. Then, we calculate the distribution of θ and v , and determined the $N_{F}$ , $\sigma_{\theta ,\;F}$ , and $\sigma_{v,\;F}$ as follows:

(76) $$N_{F}=5$$

(77) $$\sigma_{\theta ,\;F}=0.067\;rad$$

(78) $$\sigma_{v,\;F}=0.28\;m/s$$

The above values are different from those in the Kalman smoother because the observation cycle and the observation accuracy are different from those of the GNSS in the smartphone. As a result, $R_{F}$ was expressed as follows:

(79) $$R_{F}=\left(\begin{array}{cccc} \sigma_{pos,F}^{2} & 0 & 0 & 0\\ 0 & \sigma_{pos,F}^{2} & 0 & 0\\ 0 & 0 & \sigma_{\theta ,F}^{2} & 0\\ 0 & 0 & 0 & \sigma_{v,F}^{2} \end{array}\right)=\left(\begin{array}{cccc} 0.1^{2} & 0 & 0 & 0\\ 0 & 0.1^{2} & 0 & 0\\ 0 & 0 & 0.067^{2} & 0\\ 0 & 0 & 0 & 0.28^{2} \end{array}\right)$$

### 5.3. Conventional Method as a Baseline for Comparison

**Figure 21:**
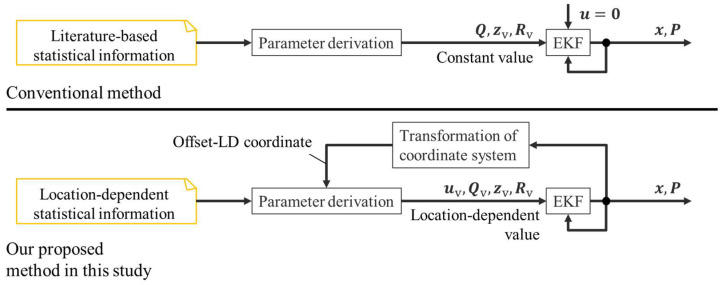
Comparison between the conventional method in our previous study and the method proposed in this study.

Although comparisons with existing methods are necessary for evaluating the proposed method in this study, only a limited number of studies were conducted in a similar situation to this study. Among the existing studies, a method that utilizes virtual observation derived from literature-based statistical information [7] was the most related and competitive to this study. Thus, we set up a conventional virtual observation method using literature-based statistical information as the baseline for comparison. Literature-based statistical information provides an average velocity that is universal for various roads and does not provide location-dependent information related to the yawing rate and acceleration. Thus, the conventional method does not use the virtual control input. Additionally, the values of the process noise matrix Q were the same as those used for the Kalman smoother. Figure 21 shows a comparison between the conventional method used in our previous study and the method proposed in this study.

As for the velocity of the conventional virtual observation, we calculated the average value and standard deviation based on the literature. Reference [32] describes that the average velocity of cyclists in Japan is approximated as 15 km/h, and the distribution of cyclists’ velocity ranges from 2.8 m/s to 6.9 m/s. Because this is not symmetrical distribution, we approximated this distribution as a normal distribution whose average velocity is 4.2 m/s and 95% confidence interval ranges from 1.5 m/s to 6.9 m/s. Consequently, the values related to the velocity of the conventional virtual observation were calculated as follows:

(80) $${\tilde{\mu }}_{v}=4.2\;m/s$$

(81) $${\tilde{\sigma }}_{v}^{\prime }=1.4\;m/s$$

As for the directional angle, there is no literature-based information. Thus, we assumed that 95% of the directional angle was distributed within a range of ±15 deg. As a result, the values related to the directional angle of the virtual observation are calculated as follows:

(82) $${\tilde{\mu }}_{\theta }=\theta_{link}$$

(83) $${\tilde{\sigma }}_{\theta }^{\prime }=0.13\;rad$$

where $\theta_{link}$ denotes the directional angle of the link. Based on the above values, we set $z_{v}$ as follows:

(84) $$z_{v}={\left(\begin{array}{cc} {\tilde{\mu }}_{\theta }\left(t_{k}\right) & {\tilde{\mu }}_{v}\left(t_{k}\right) \end{array}\right)}^{T}={\left(\begin{array}{cc} \theta_{link} & 4.2 \end{array}\right)}^{T}$$

Additionally, we calculated the observation noise matrix $R_{v}$ for the conventional method based on Equations (69)–(71) with ${\tilde{\sigma }}_{v}^{\prime }$ and ${\tilde{\sigma }}_{\theta }^{\prime }$ . The conventional method estimates the position of cyclists in an observation cycle of 1.0 Hz using virtual observation.

### 5.4. Results and Discussions

#### 5.4.1. Position Estimation Results for Representative Cases

**Figure 22:**
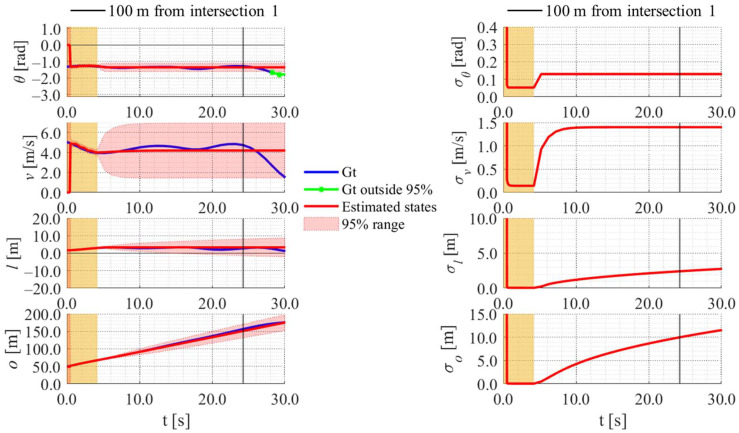
Estimated results of data 22 in Cluster 2 using the conventional method.

**Figure 23:**
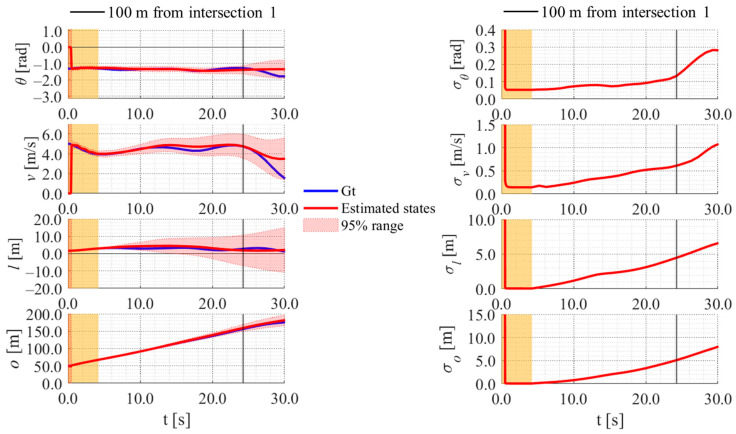
Estimated results of data 22 in Cluster 2 using the proposed method.

**Figure 24:**
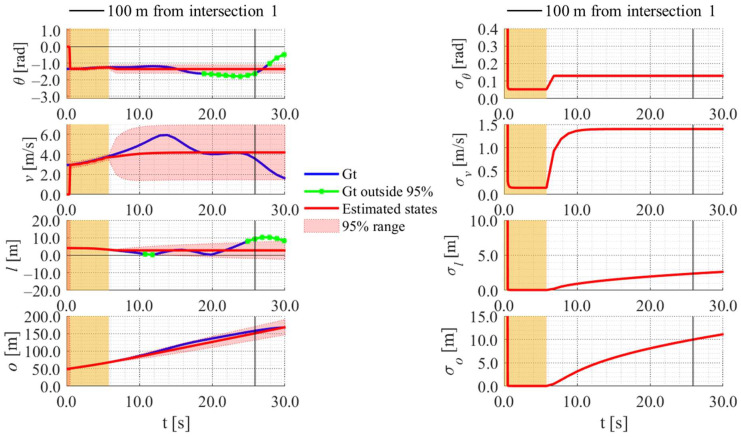
Estimated results of data 11 in Cluster 2 using the conventional method.

**Figure 25:**
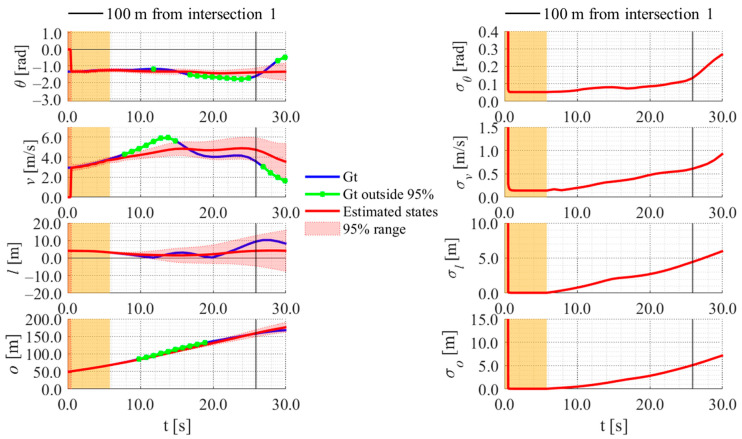
Estimated results of data 11 in Cluster 2 using the proposed method.

Figure 22, Figure 23, Figure 24 and Figure 25 show the estimation results of data 22 by the conventional method, those by the proposed method, the estimation results of data 11 by the conventional method, and those by the proposed method, respectively. In each figure, the left graph shows the estimated variables, 95% confidence interval, and ground truth values. The orange areas around 0.0 s indicate the duration during which the roadside sensor observed the cyclists. Red lines indicate the estimated variables, and red areas indicate 95% confidence intervals. Blue lines indicate ground truth values. The green dots indicate the ground truth values that were outside the 95% confidence interval. The graph on the right shows the time-series uncertainty related to each variable. As for the characteristics of the data, data 22 branched out in the relatively lower area of the dendrogram shown in Figure 16, indicating that the data of this trial was located in the relatively central area of the cluster. In contrast, data 11 branched out in the relatively upper area of the dendrogram, indicating that the data of this trial was located in the relatively outlier area of the cluster.

In the results of the conventional method using literature-based statistical information, as shown in Figure 22, the estimated values of the directional angle and velocity converged to the average values that were used for virtual observation. In addition, their uncertainty also converged to the uncertainty of the virtual observation.

In contrast, in the result of the proposed method using LDSI, as shown in Figure 23, the estimated velocity changed at each location. Additionally, the uncertainty of the estimated directional angle and velocity changed depending on the uncertainty of the LDSI. However, because the uncertainty of position estimation affected the uncertainty of the virtual observation, as described in Section 3.4.3, the uncertainty of the other estimated variables increased according to the increase in the uncertainty of the position estimation because of the long time since the roadside observation. Furthermore, in the case where the ground truth of velocity was outside the 95% confidence interval, as shown in Figure 25, the ground truth of position was also outside the 95% confidence interval. Because data 11 was located in the relatively outlier area of the cluster, the ground truth values might be outside the 95% confidence interval.

#### 5.4.2. Evaluation Results for All Cases

Because the final goal of this study is to prevent crossing collisions, estimation of the offset, which directly contributes to the collision prediction, is most important. From this viewpoint, although the directional angle and LD are not directly related to the offset, velocity is directly related to the offset. Therefore, in the following analyses, we focus only on velocity and offset.

Table 1 and Table 2 show the average ratios of the ground truth velocity and offset within the 95% confidence interval, respectively. First, we calculated the ratio of time-series variables inside the confidence interval from the time when the cyclist exited the observation range of the roadside sensor to the time when the cyclist arrived at the point 100 m away from the center of intersection 1 in each trial. We then calculated the average ratio from all the results. As shown in Table 1 and Table 2, although the ratio of the conventional method was better than that of the proposed method, appropriateness of the estimated values by the conventional method was not the reason; rather, the large uncertainty of the conventional method appeared to be the reason for this result.

**Figure 26:**
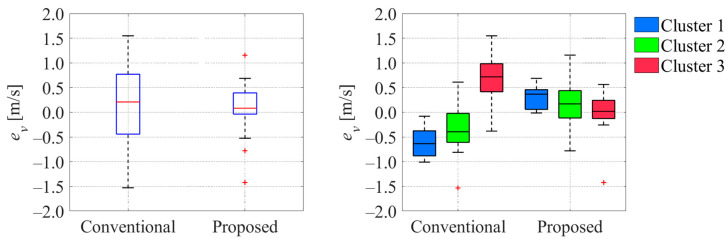
Comparison of estimated velocity error between conventional and proposed methods. The blue boxes in the left graph indicate the interquartile range, and the red lines inside the blue box indicate the median. The red plus signs in the graphs indicate the outliers.

**Figure 27:**
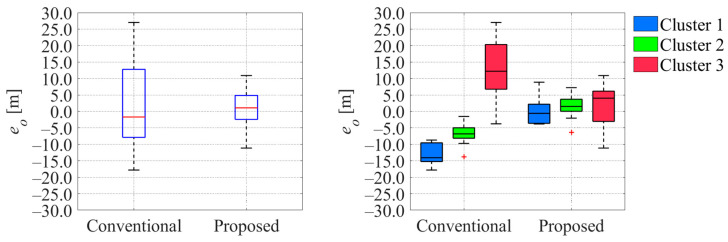
Comparison of estimated offset error between conventional and proposed methods. The blue boxes in the left graph indicate the interquartile range, and the red lines inside the blue box indicate the median. The red plus signs in the graphs indicate the outliers.

Figure 26 and Figure 27 show the comparison of the error of each variable between the ground truth values and estimated values when the cyclist reached a point 100 m ahead of the center of intersection 1. Here, $e_{v}$ and $e_{o}$ indicate the errors in velocity and offset, respectively. From Figure 26 and Figure 27, we can confirm that the errors in the estimated velocity and offset in the proposed method were smaller than those in the conventional method. Additionally, a comparison of the corresponding results in each cluster indicates that the median values in the proposed method existed around zero, and those in the conventional method were far from zero.

**Figure 28:**
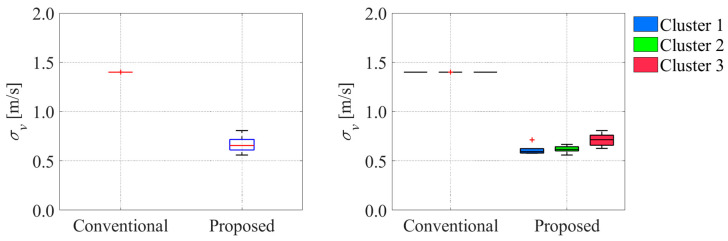
Comparison of velocity uncertainty between conventional and proposed methods. The blue boxes in the left graph indicate the interquartile range, and the red lines inside the blue box indicate the median. The red plus signs in the graphs indicate the outliers.

**Figure 29:**
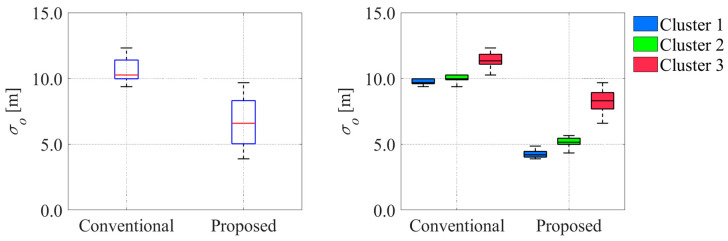
Comparison of offset uncertainty between conventional and proposed methods. The blue boxes in the left graph indicate the interquartile range, and the red lines inside the blue box indicate the median.

Similarly, Figure 28 and Figure 29 show the comparison of the uncertainty of estimated variables at the same moment. $\sigma_{v}$ and $\sigma_{o}$ indicate the uncertainties in the estimated velocity and offset, respectively. In the conventional method, the uncertainties of the estimated velocity converged to the same values as in the literature-based statistical information. By contrast, because the uncertainty of the estimated velocity depended on the uncertainty of the estimated position in the proposed method, the uncertainty of the velocity differed from each trial. In addition, the uncertainties of the offset in the proposed method were smaller than those in the conventional method.

#### 5.4.3. Discussion

To evaluate the position estimation methods, discussions from the perspectives of adequacy and performance are necessary. Regarding adequacy, the average ratio of the ground truth variables inside the 95% confidence interval were 93% and 91% in the proposed method with ideal classification; they were less than 95%. As shown in Figure 25, the cases in which acceleration/deceleration was more intense than average show a low ratio of the ground truth value inside the confidence interval. Such trials are basically clustered in the upper area of the dendrogram shown in Figure 16, which indicates that such trials are relatively outliers in the cluster. Thus, if we accumulate much more data and increase the number of clusters, such trials will belong to the new clusters; further, it will increase the average ratio inside the confidence interval and improve the adequateness.

As for the performance, although the estimated values in the conventional method converged to constant values based on literature-based statistical information, those in the proposed method changed according to the location. As a result, the proposed method could decrease the errors of the estimated values when the cyclist arrived at the point that was 100 m away from the roadside sensor in intersection 1. Here, the median errors of each cluster estimated by the proposed method were better than those obtained by the conventional method. Thus, consideration of movement characteristics from the viewpoint of group differences may contribute to the improvement of the results. Furthermore, the smaller uncertainty of the estimated velocity in the proposed method also contributes to the improvement in the uncertainty of the offset, which will contribute to the adequate behaviors of connected ADASs [6]. From these results, we can confirm that the proposed method improved the performance of movement estimation owing to the use of LDSI.

### 5.5. Position Estimation Results with Miss Classification

In the above estimation results, we assumed an ideally accurate classification method, as mentioned in Section 5.1. However, realization of such ideal classification method is practically hard, and miss-classifications are inevitable in the real conditions to a certain degree. Therefore, we also simulated position estimation with wrong classifications, and analyzed the average ratio within the 95% confidence interval, similar to Section 5.4.2. Table 3 shows the comparison of the average ratio between estimation with correct classifications and wrong classifications. In general, the average ratios within the 95% confidence interval with wrong classification are low compared to those with correct classification. In detail, because the movement characteristics of each cluster are different from each other, the degree of degrading the ratio is various.

Because the results indicate the importance of correct classification, development of an accurate classification method will be required. Additionally, not only the high accuracy of the classification method but also consideration of classification uncertainty are important. In particular, to prevent inappropriate position estimation with large errors caused by wrong classification, position estimation method considering the classification uncertainty is important. For instance, when the uncertainty of the classification is large, utilization of the literature-based statistical information instead of LDSI would contribute to the improvement of estimation robustness. Such method, which switches the estimation method to a conservative one based on classification uncertainty can be one of the solutions to improve robustness of proposed method.

### 5.6. Limitations and Future Prospects

Because this study was conducted as a first step of the proposed approach, we assumed many ideal conditions. First, we conducted position estimation only with the simulation. Thus, the feasibility validation of proposed method was limited. It is desirable to use the data obtained by actual roadside sensors for validation. Additionally, it is also desirable to use the data with precise ground truth value. Therefore, an experiment in the real-world using actual roadside sensors as well as real time kinematic GNSS for obtaining ground truth values is necessary. Such actual data will enable us to validate proposed method in detail.

Second, we used the estimated trajectories by Kalman smoother as the ground truth in the simulation. Utilization of the estimated trajectories, which are the original sources of LDSI, as the ground truth may increase the risk of inflating the performance in the simulation. However, virtual observation and virtual control input are not calculated directly from the ground truth data. They are calculated from LDSI, which is not a value directly related to a single data, but a representative value of multiple data. Thus, the risk of inflating the performance was not crucial. However, although the risk was limited, additional data is necessary to separate data for preparing LDSI and those for simulation to conduct more appropriate verification.

Third, we assumed static environmental conditions, and did not consider the change in the cyclists’ movement caused by changes in the environmental conditions, such as time of day, weather, and so on. Thus, robustness of proposed method to the changes in environmental conditions was limited. To overcome such limitation, a detection method for the changes in environmental conditions and an adaptation method of LDSI to such changes are required. In the future smart city, some cyclists are assumed to be real-time connected to the system. The real-time information including movement and density from such cyclists enables the system to detect the changes in environmental conditions. When the changes in environmental conditions are detected, the system can adapt LDSI to them. Position estimation with such adaptive LDSI will improve the robustness of proposed method.

Finally, in this study, only 10 participants were recruited to the experiment, and the generality of data was limited. To overcome such limitation, utilization of GNSS data of smartphones collected by telecommunication companies or smartphone-application companies is effective. A study using such data for analysis of pedestrians’ travel characteristics in macro scale [38] was already conducted. Although existing study [38] conducted macroscopic people-flow analysis, applying the proposed method in this study to such data will contribute to the microscopic people-flow analysis. In addition, because the study [38] demonstrated that the large amount of GNSS data is available from such data sources, sufficient data for constructing general LDSI would be available. Furthermore, considering the high penetration ratio of smartphones, GNSS data are collected from wide range of people. Therefore, we think that sufficient data from various cyclists will reflect the actual characteristics of the cyclists on the roads where corresponding data are obtained. Thus, the utilization of such data enables a more general validation of the proposed method.

## 6. Conclusions

In this study, we developed a method for preparing LDSI from accumulated GNSS data of cyclists using the Kalman smoother. We then developed a movement estimation method using virtual observation and virtual control input based on LDSI. To evaluate the proposed methods, we conducted an experiment to accumulate the GNSS data of cyclists. From the experimental data, we confirmed that our proposed method adequately prepared the LDSI. In addition, we confirmed that our proposed method using LDSI estimated the movement state of cyclists better than the conventional method. These methods will contribute to the prevention of cyclist-related crossing collisions around nonsignalized intersections with occlusions on community roads, where roadside sensors are sparsely placed.

However, further studies are still needed. In this study, because this study aimed to develop the methods as a first step, the number of the experimental participants and the variations was limited. Thus, increasing the number of participants for data accumulation and the variations in traffic environments is necessary. Additionally, we simulated a numerical experiment of the position estimation of cyclists with the assumption of an ideal classification system that classifies cyclists into appropriate clusters related to LDSI. Thus, we need to develop a classification system based on information from roadside sensors. Additionally, real-world experiments on actual community roads need to be conducted using actual roadside sensors. After completing the above future works, we will try to implement the proposed method in this study into the connected ADASs including motion control.

## Figures and Tables

**Table 1:** Average ratio within the 95% confidence interval in velocity estimation.

Methods	All Data (29 Data)	Cluster 1 (5 Data)	Cluster 2 (9 Data)	Cluster 3 (15 Data)
Conventional	1.00	1.00	1.00	1.00
Proposed	0.93	0.90	0.92	0.94

**Table 2:** Average ratio within the 95% confidence interval in offset estimation.

Methods	All Data (29 Data)	Cluster 1 (5 Data)	Cluster 2 (9 Data)	Cluster 3 (15 Data)
Conventional	0.97	1.00	1.00	0.95
Proposed	0.91	0.77	0.93	0.93

**Table 3:** Average ratio within 95% confidence interval in the state estimation with wrong classification.

GT Cluster		Cluster 1 (5 Data)			Cluster 2 (9 Data)			Cluster 3 (15 Data)		
Classified Cluster		1	2	3	1	2	3	1	2	3
		Correct	Wrong	Wrong	Wrong	Correct	Wrong	Wrong	Wrong	Correct
Average ratio within the confidence interval	Velocity	0.90	0.72	0.38	0.65	0.92	0.89	0.44	0.46	0.94
	Offset	0.77	0.65	0.36	0.57	0.93	0.83	0.24	0.15	0.93

## Data Availability

The original contributions presented in this study are included in the article.

## Abbreviations

The following abbreviations are used in this manuscript:

GNSS	Global navigation satellite system
VRU	Vulnerable road user
ADAS	Advanced driver-assistance system
LDSI	Location-dependent statistical information
RSU	Roadside unit
V2P	Vehicle to pedestrian
V2I	Vehicle to infrastructure
P2I	Pedestrian to infrastructure
ML	Machine learning
LSTM	Long short-term memory
EKF	Extended Kalman filter
RNN	Recurrent neural network
NN	Neural network
LD	Lateral deviation
SWLDSI	Stochastically weighted location-dependent statistical information

## Appendix A

The derivations of Equation (53) in Section 3.4.3 are as follows:

(A1)
$$\begin{array}{cc} & {\tilde{\mu }}_{v}\left(t\right)=E\left(v\right)\\ & =\int_{-\infty }^{\infty }vp_{SW}\left(v,t\right)dv\\ & =\int_{-\infty }^{\infty }v\sum_{i=1}^{n}\alpha_{i}\left(t\right)\frac{1}{\sqrt{2\pi {\tilde{\sigma }}_{LDSIv,i}^{2}}}\exp\left[-\frac{{\left(v-{\tilde{\mu }}_{LDSIv,i}\right)}^{2}}{2{\tilde{\sigma }}_{LDSIv,i}^{2}}\right]dv \end{array}$$

Here, the first two derivations are from definition, and the last derivation is obtained by Equation (52). The rest of the derivation is as follows:

(A2)
$$\begin{array}{cc} & {\tilde{\mu }}_{v}\left(t\right)=\sum_{i=1}^{n}\alpha_{i}\left(t\right)\int_{-\infty }^{\infty }v\frac{1}{\sqrt{2\pi {\tilde{\sigma }}_{LDSIv,i}^{2}}}\exp\left[-\frac{{\left(v-{\tilde{\mu }}_{LDSIv,i}\right)}^{2}}{2{\tilde{\sigma }}_{LDSIv,i}^{2}}\right]dv\\ & =\sum_{i=1}^{n}\alpha_{i}\left(t\right){\tilde{\mu }}_{LDSIv,i} \end{array}$$

The first derivation can be obtained because $\alpha_{i}\left(t\right)$ is not a function of v . The last derivation is obtained from the definition of normal distribution.

The derivations of Equation (54) in Section 3.4.3 are as follows:

(A3)
$$\begin{array}{cc} & {\tilde{\sigma }}_{v}^{2}\left(t\right)=Var\left(v\right)\\ & =E\left(v^{2}\right)-{\left[E\left(v\right)\right]}^{2}\\ & =\int_{-\infty }^{\infty }v^{2}p_{SW}\left(v,\;t\right)dv-{\left[\sum_{i=1}^{n}\alpha_{i}\left(t\right){\tilde{\mu }}_{LDSIv,i}\right]}^{2}\\ & =\int_{-\infty }^{\infty }v^{2}\sum_{i=1}^{n}\alpha_{i}\left(t\right)\frac{1}{\sqrt{2\pi {\tilde{\sigma }}_{LDSIv,i}^{2}}}\exp\left[-\frac{{\left(v-{\tilde{\mu }}_{LDSIv,i}\right)}^{2}}{2{\tilde{\sigma }}_{LDSIv,i}^{2}}\right]dv-{\left[\sum_{i=1}^{n}\alpha_{i}\left(t\right){\tilde{\mu }}_{LDSIv,i}\right]}^{2} \end{array}$$

Here, the first two derivations are from definition, and the third derivation is obtained by using Equation (A2). The last derivation is obtained by Equation (52). The rest of the derivations are as follows:

(A4)
$$\begin{array}{cc} & {\tilde{\sigma }}_{v}^{2}\left(t\right)=\sum_{i=1}^{n}\alpha_{i}\left(t\right)\int_{-\infty }^{\infty }v^{2}\frac{1}{\sqrt{2\pi {\tilde{\sigma }}_{LDSIv,i}^{2}}}\exp\left[-\frac{{\left(v-{\tilde{\mu }}_{LDSIv,i}\right)}^{2}}{2{\tilde{\sigma }}_{LDSIv,i}^{2}}\right]dv-{\left[\sum_{i=1}^{n}\alpha_{i}\left(t\right){\tilde{\mu }}_{LDSIv,i}\right]}^{2}\\ & =\sum_{i=1}^{n}\alpha_{i}\left(t\right)\left({\tilde{\sigma }}_{LDSIv,i}^{2}+{\tilde{\mu }}_{LDSIv,i}^{2}\right)-{\left[\sum_{i=1}^{n}\alpha_{i}\left(t\right){\tilde{\mu }}_{LDSIv,i}\right]}^{2}\\ & =\sum_{i=1}^{n}\alpha_{i}\left(t\right){\tilde{\sigma }}_{LDSIv,i}^{2}+\sum_{i=1}^{n}\alpha_{i}\left(t\right){\tilde{\mu }}_{LDSIv,i}^{2}-{\left[\sum_{i=1}^{n}\alpha_{i}\left(t\right){\tilde{\mu }}_{LDSIv,i}\right]}^{2} \end{array}$$

The first derivation can be obtained because $\alpha_{i}\left(t\right)$ is not a function of v . The second derivation is obtained by using the definition of normal distribution.
