# Correction: Suzuki, K.; Ito, T. Virtual Observation Using Location-Dependent Statistical Information of Cyclists’ Movement for Estimation of Position and Uncertainty. *Sensors* 2025, *25*, 5122

**DOI:** 10.3390/s26020697

**Published:** 2026-01-21

**Authors:** Kento Suzuki, Takuma Ito

**Affiliations:** Graduate School of Engineering, The University of Tokyo, 7-3-1 Hongo, Tokyo 113-8656, Japan

An implementation mistake was found in our simulation program which makes minor changes in some figures and a table in the original publication [1].

## Figure Correction

In the original publication [1], there were mistakes in Figures 23 and 25–29. The corrected version of the figures are provided below.

**Figure 23:**
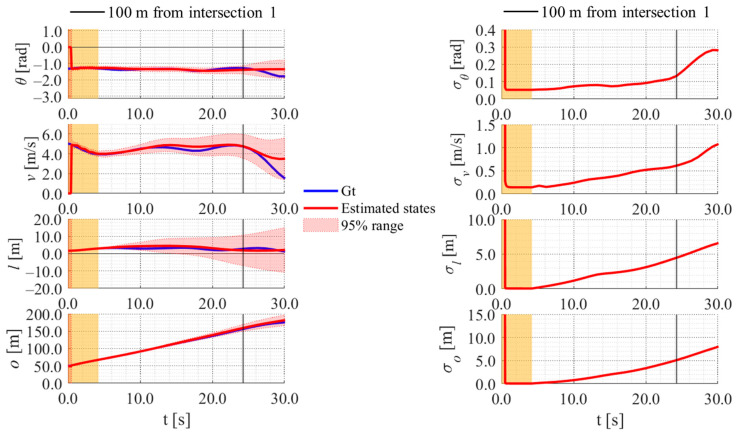
Estimated results of data 22 in Cluster 2 using the proposed method.

**Figure 25:**
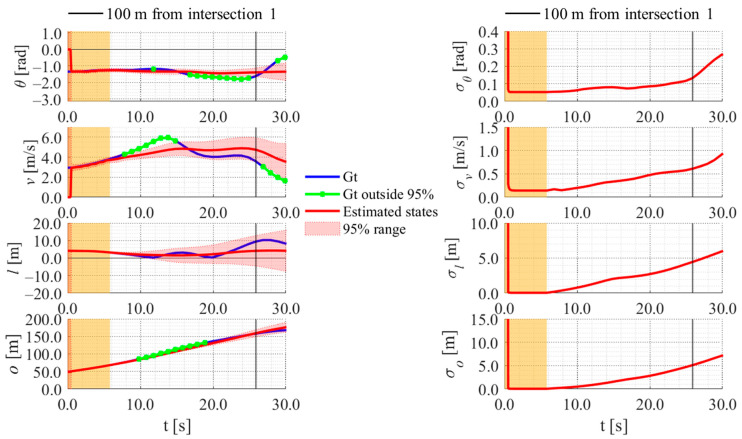
Estimated results of data 11 in Cluster 2 using the proposed method.

**Figure 26:**
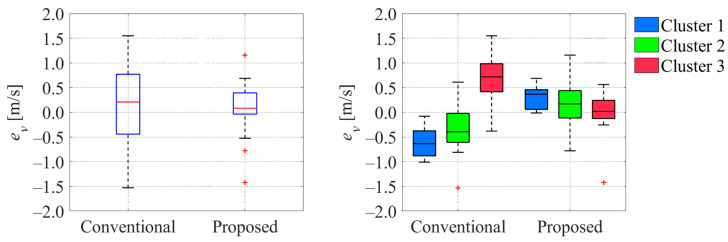
Comparison of estimated velocity error between conventional and proposed methods. The blue boxes in the left graph indicate the interquartile range, and the red lines inside the blue box indicate the median. The red plus signs in the graphs indicate the outliers.

**Figure 27:**
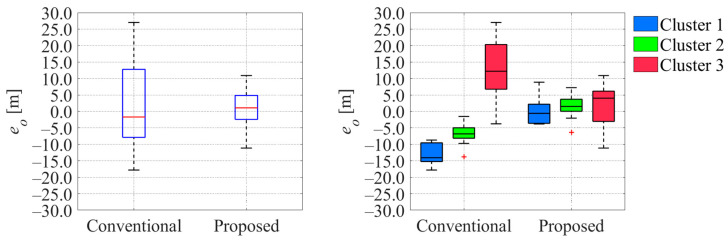
Comparison of estimated offset error between conventional and proposed methods. The blue boxes in the left graph indicate the interquartile range, and the red lines inside the blue box indicate the median. The red plus signs in the graphs indicate the outliers.

**Figure 28:**
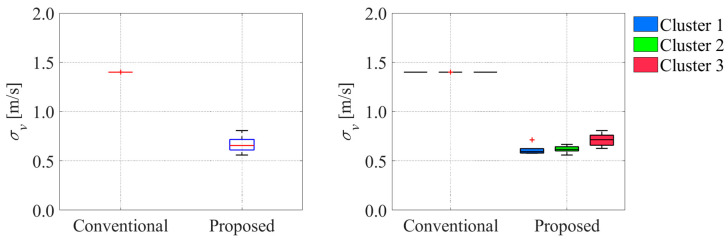
Comparison of velocity uncertainty between conventional and proposed methods. The blue boxes in the left graph indicate the interquartile range, and the red lines inside the blue box indicate the median. The red plus signs in the graphs indicate the outliers.

**Figure 29:**
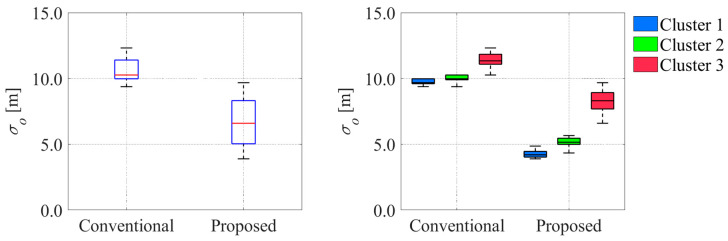
Comparison of offset uncertainty between conventional and proposed methods. The blue boxes in the left graph indicate the interquartile range, and the red lines inside the blue box indicate the median.

## Table Correction

In the original publication [1], there were mistakes in Table 3. The corrected version of the table is provided below. 

**Table 3:** Average ratio within 95% confidence interval in the state estimation with wrong classification.

GT Cluster		Cluster 1 (5 Data)			Cluster 2 (9 Data)			Cluster 3 (15 Data)		
Classified Cluster		1	2	3	1	2	3	1	2	3
		Correct	Wrong	Wrong	Wrong	Correct	Wrong	Wrong	Wrong	Correct
Average ratio within the confidence interval	Velocity	0.90	0.72	0.38	0.65	0.92	0.89	0.44	0.46	0.94
	Offset	0.77	0.65	0.36	0.57	0.93	0.83	0.24	0.15	0.93

The authors apologize for any inconvenience caused and state that the scientific conclusions are unaffected. The original article has been updated. This correction was approved by the Academic Editor. The original publication has also been updated.

## References

[1] Suzuki K., Ito T. (2025). Virtual Observation Using Location-Dependent Statistical Information of Cyclists’ Movement for Estimation of Position and Uncertainty. Sensors.

